# Impact of Diabetes and Metformin on Cardiovascular Outcomes in Prostate Cancer Patients Aged 66 and Older: The Role of Social Determinants of Health and Racial Disparities †

**DOI:** 10.3390/cancers17172854

**Published:** 2025-08-30

**Authors:** Priyanshu Nain, Omar M. Makram, Viraj Shah, Harikrishnan Hyma Kunhiraman, Nickolas Stabellini, Biplab Datta, Stephanie Jiang, Vraj Patel, Lakshya Seth, Aditya Bhave, Sarah A. Malik, Yan Gong, Michael G. Fradley, Darryl P. Leong, Ryan A. Harris, Yi-Hsin Hung, Austin Yen-Hung Lin, Neal L. Weintraub, Avirup Guha

**Affiliations:** 1 Department of Internal Medicine, Advent Health, Rome, GA 30165, USA; pnain@augusta.edu; 2 Division of Cardiology, Department of Medicine, Medical College of Georgia, Augusta University, Augusta, GA 30912, USA; omelsayed@augusta.edu (O.M.M.); vishah@augusta.edu (V.S.); hhymakunhiraman@augusta.edu (H.H.K.); nweintraub@augusta.edu (N.L.W.); 3 Cardio-Oncology Program, Medical College of Georgia, Augusta University, Augusta, GA 30912, USA; 4 Department of Hematology-Oncology, University Hospitals Seidman Cancer Center, Cleveland, OH 44106, USA; nstabellini@augusta.edu; 5 Institute of Public and Preventive Health, Augusta University, Augusta, GA 30912, USA; bdatta@augusta.edu; 6 Department of Neurology, Perelman School of Medicine, University of Pennsylvania, Philadelphia, PA 19104, USA; stephanie.jiang@pennmedicine.upenn.edu; 7 Department of Internal Medicine, Heersink School of Medicine, University of Alabama at Birmingham, Birmingham, AL 35233, USA; vrapatel@augusta.edu; 8 Department of Internal Medicine, University of Texas Southwestern Medical Centre, Dallas, TX 75390, USA; lseth@augusta.edu; 9 Medical College of Georgia, Augusta University, Augusta, GA 30912, USA; abhave@augusta.edu; 10 Department of Internal Medicine, Duke University Medical Centre, Durham, NC 27710, USA; sarah.malik@duke.edu; 11 College of Pharmacy, University of Florida, Gainesville, FL 32611, USA; gong@cop.ufl.edu; 12 Division of Cardiology, Department of Medicine, Thalheimer Center for Cardio-Oncology, Perelman School of Medicine, University of Pennsylvania, Philadelphia, PA 19104, USA; michael.fradley@pennmedicine.upenn.edu; 13 The Population Health Research Institute, McMaster University and Hamilton Health Sciences, 237 Barton St. East, Hamilton, ON L8L 2X2, Canada; darryl.leong@phri.ca; 14 Georgia Prevention Institute, Medical College of Georgia, Augusta University, Augusta, GA 30912, USA; ryharris@augusta.edu; 15 Division of Cardiology, Department of Internal Medicine, National Taiwan University Hospital, Taipei 100229, Taiwan; a03144@ntucc.gov.tw (Y.-H.H.); yenhunglin@ntuh.gov.tw (A.Y.-H.L.); 16 Cardiovascular Center, National Taiwan University Hospital, Taipei 100229, Taiwan

**Keywords:** cardio-oncology, diabetes, prostate cancer, SDOH, disparities

## Abstract

**Background:** This study evaluated the impact of diabetes mellitus (DM) and its treatments on cardiovascular outcomes in prostate cancer (PC) patients aged 66 years and older, with or without androgen deprivation therapy (ADT) exposure. **Methods:** Using the SEER-Medicare database (2009–2017), two cohorts were created: Cohort 1 included all PC patients enrolled in Medicare Parts A and B; Cohort 2 was a subset of Cohort 1 receiving ADT and enrolled in Medicare Part D. Exposures were DM and DM medications. Outcomes included cardiovascular events (CVEs), cardiovascular mortality (CVm), PC-specific mortality (PCsm), and all-cause mortality, analyzed using multivariable Fine-Gray and Cox models. **Results:** Cohort 1 included 150,647 PC patients (32% with DM, median age 72). DM was associated with higher risk of CVE (subdistribution hazard ratio [sHR] 1.20, 95% CI 1.17–1.22), CVm (sHR 1.35, 1.28–1.43), and all-cause mortality (adjusted HR [aHR] 1.22, 1.19–1.26) (all *p* < 0.001). Non-Hispanic Blacks (NHBs) and patients from lower socioeconomic (SES) and education areas experienced comparable or worse outcomes. In Cohort 2 (*n* = 14,938), DM patients on non-metformin therapies had higher all-cause mortality (aHR 1.33, 1.11–1.25; *p* = 0.002) than those on metformin, particularly in NHB and low education groups. Sensitivity analyses with follow-up limited to two years showed consistent results as overall. **Conclusions:** Diabetic PC patients, especially NHB, lower SES and lower education subgroups, were associated with worse cardiovascular and all-cause mortality outcomes. Metformin may be associated with better outcomes in these populations, warranting further research on the disparities in PC and diabetes, and cardioprotective effects of DM medications across different subpopulations.

## Simple Summary

This study looked at how diabetes affects the risk of heart disease and death in older prostate cancer patients, especially among those from underserved communities. The results showed that men with both diabetes and prostate cancer had a higher risk of heart-related illness and death compared to those without diabetes. These risks were especially high for Black men and people living in neighborhoods with low income or education levels. However, among patients taking hormone therapy for their prostate cancer, those who were also treated with metformin had better survival rates. This suggests that metformin may help protect the heart and improve outcomes in these high-risk groups.

## 1. Introduction

Diabetes mellitus (DM) is a common comorbidity in older men and has a complex relationship with prostate cancer (PC). Epidemiologic studies suggest that DM is associated with a modest reduction in PC incidence; however, once diagnosed, men with DM often experience worse cancer-specific and overall survival [1,2,3,4]. These effects have been attributed to changes in sex hormone levels, insulin/IGF signaling, chronic inflammation, and other metabolic alterations that can influence PC biology and progression [5,6,7]. Importantly, this relationship is observed even in men who have not received androgen deprivation therapy (ADT), underscoring that DM may affect PC outcomes through mechanisms related to disease biology, independent of treatment exposure [8,9].

Advances in prostate cancer (PC) treatment have shifted the burden of mortality toward cardiovascular disease (CVD). CVD now affects nearly two-thirds of men with PC and has surpassed the malignancy itself as the leading cause of death in this population [10,11]. Recent SEER-based data places PC among the top cancer types for both early and late cardiovascular mortality, emphasizing the need for targeted cardio-oncology strategies in this highly prevalent group [12,13].

Despite the well-documented interplay between DM and PC, and increasing CVD burden, most studies have evaluated outcomes in terms of only cancer incidence and mortality, rarely considering CVD as a primary endpoint [13]. This omission is also notable because PC patients already face a higher metabolic risk profile than survivors of other cancers and the general population, further amplifying their vulnerability to CVD [7,14,15]. While prior research has examined CVD risk in diabetic patients on ADT [16,17,18], little is known about cardiovascular outcomes, especially the incidence of cardiovascular disease in metabolically abnormal PC patients more broadly [19,20]. Addressing this gap is critical to understanding the full impact of DM in PC and identifying high-risk subgroups for targeted cardio-oncology interventions.

Androgen-deprivation therapy (ADT) is consistently linked to higher cardiovascular risk and worsened glycemic control, including higher rates of diabetes, metabolic syndrome features, and heart failure [21,22,23]. There is no established pharmacologic cardioprotection for men on ADT; management largely relies on risk-factor control and, at best, selection of lower-risk hormonal strategies (e.g., GnRH antagonists vs. agonists) rather than a dedicated agent [24]. This lack of cardioprotective options is especially consequential for patients with pre-existing diabetes, because ADT can aggravate insulin resistance and dyslipidemia, compounding cardiovascular risk [25,26,27]. Metformin, an agent with emerging antitumor evidence in PC and well-described metabolic benefits, has recently been shown to improve weight, glycemic control, and lipid profiles during ADT, raising the question of whether it may also translate into improved outcomes, especially cardiovascular, in diabetic men receiving ADT [27,28]. Metformin use has been associated with some improvements in overall survival and PC-specific mortality [29,30]. However, its impact on CVD in diabetic patients with PC, in the presence of ADT, is unknown.

Moreover, health disparities among non-Hispanic Blacks (NHBs) and various social determinants of health (SDOH), including education, socioeconomic status (SES), and rurality, have been shown to significantly impact PC survival and further complicate the relationship between DM and CV outcomes in these patients [6,31,32]. In this Surveillance, Epidemiology, and End Results (SEER)-Medicare registry study, we aimed to analyze the impact of DM and its treatments on the incidence of various CV outcomes in PC patients with or without a history of ADT. We also examined the relationship between NHB individuals and those living in neighborhoods with low SES and low education levels.

## 2. Materials and Methods

We utilized the linked SEER-Medicare databases spanning from 2009 to 2017, with the latest relevant linkage completed in 2022. While the latest linkage is the 2022 linkage, the investigators received data from the 2020 linkage. The SEER program, endorsed by the National Cancer Institute (NCI), Bethesda, MD, USA, encompasses 18 statewide or regional cancer registries, collectively covering approximately 47.9% of the US population [33]. It systematically gathers data on patient demographics, cancer characteristics, and survival outcomes for individuals diagnosed with cancer within their respective catchment areas. The SEER-Medicare linkage provides a comprehensive dataset combining cancer registry information with healthcare utilization, cost, and treatment data for U.S. patients aged 65 and older. It includes data from Medicare Parts A (hospital care), B (outpatient and hospital physician services), and D (prescription drugs), offering detailed insight into baseline characteristics, comorbidities, and clinical outcomes (Centers for Medicare & Medicaid Services, Baltimore, MD, USA). Detailed insights into Medicare Parts can be found at Centers for Medicare & Medicaid Services website [34].

### 2.1. Study Design and Cohort

We utilized a retrospective cohort study design. A PC diagnosis was established using the International Classification of Diseases for Oncology, Third Edition (ICD-O-3), site recode classification C61. Two cohorts of men aged 65 years or older diagnosed with primary PC between 2009 and 2017 were established. Cohort 1 included all eligible aged 66 years and above PC patients enrolled in both Medicare Parts A and B. This cohort was created to study the overall effect of DM on various outcomes, including CVE, CVm, PCsm, and all-cause mortality. Cohort 2 was created as a subset of individuals from Cohort 1, additionally requiring enrollment in Medicare Part D and documented the receipt of ADT treatment (defined below). Cohort 2 was created specifically to investigate the relationship between different DM mediations (metformin vs. others) and the outcomes among the PC patients who received ADT. DM diagnosis was based on ICD codes 250.xx and 362.0x, as well as chronic condition flags.

Any patient in both cohorts with missing follow-up or cancer diagnosis dates was excluded from the analysis (Supplementary Table S16).

### 2.2. Exposure

For cohort 1, the primary exposure of interest was DM. In cohort 1, we further supplemented the DM data from the chronic condition flag file. For cohort 2, the exposure of interest was the utilization of metformin alone or in combination with other medications versus the use of other DM medications. Using Part-D Medicare (Centers for Medicare & Medicaid Services, Baltimore, MD, USA) claims files, DM medications identified were metformin, insulin, and other medications (i.e., alpha-glucosidase inhibitors, amylin analogs, dipeptidyl peptidase-4 (DPP-4) inhibitors, meglitinides, sulfonylureas, thiazolidinediones) based on Food and Drug Administration (FDA) approval until 2019. For the purpose of this analysis, those who used metformin alone or in combination with other DM medications were considered as individuals with DM in the metformin group. Those using medications other than metformin were considered as individuals with DM in the other medications group. This grouping aligns with prior studies and reflects real-world prescribing practices [35,36]. Further explanation and details for the ICD-9 and -10 used for defining the exposure and outcomes variables are provided in Supplementary Table S1.

### 2.3. Outcomes

Patients were followed from PC diagnosis until 31 December 2019, for all outcomes reported in the study. In this analysis, our primary outcome was CVE, defined as developing heart failure (HF), atrial fibrillation (AF), acute myocardial infarction (MI), peripheral artery disease (PAD), or ischemic stroke (IS) during the entire follow-up period. To enhance the accuracy and comprehensiveness of CVE identification, we also incorporated data from the chronic condition flag file. This segment tracks 27 chronic conditions, including CVE, providing a robust framework for our analysis. If a CVE diagnosis date appeared in more than one source, then the earliest date of diagnosis was used. Those who were determined to have CVE before cancer diagnosis were considered to have a prior history of CVD and excluded. Secondary outcomes included CVm, PCsm, and all-cause mortality. CVm included all causes of death related to any circulatory system diseases, and was defined using the ICD-10 code (I00-I99) (Supplementary Table S1) [37].

### 2.4. Covariates

Covariates collected at baseline included age at cancer diagnosis, race/ethnicity (non-Hispanic White [NHW], NHB, Hispanic, and other), marital status (single, married, other, and unknown), SDOH including SES (high and low), education level (high and low), and rurality (rural and urban). The other race/ethnicity was defined as being non-Hispanic American Indian, Alaska Native, Asian, or Pacific Islander. Other variables included prostate cancer characteristics, including American Joint Committee on Cancer (AJCC) staging and grade, prostate cancer treatment (surgery, radiotherapy, and ADT), and past medical history of hypertension, hyperlipidemia, and chronic kidney disease (CKD). The past medical history variables were obtained from the Medicare chronic condition files. For ADT definition, we included all the following treatments: apalutamide (Janssen Biotech, Horsham, PA, USA), bicalutamide (Pfizer Inc., New York, NY, USA), darolutamide (Bayer AG, Whippany, NJ, USA), degarelix acetate (Ferring Pharmaceuticals, Parsippany, NJ, USA), enzalutamide (Astellas Pharma US, Northbrook, IL, USA), flutamide (Schering Corporation, Kenilworth, NJ, USA), goserelin acetate (AstraZeneca Pharmaceuticals, Wilmington, DE, USA), and leuprolide acetate (AbbVie Inc., North Chicago, IL, USA). Socioeconomic status was defined at the census-tract level using the Yost index (a measure for SES that includes factors such as education, income, housing, and employment), where those living in areas within the first and second quintiles were considered low SES and any others were considered high SES [38]. Education level was defined at the county/parish level using the Federal Information Processing System (FIPS) Codes for States and Counties, where those living in areas with high school diplomas of less than 25% were considered living in low education level areas [39]. Rurality was assessed at the county level, and we used the 2013 Rural-Urban Continuum Codes (RUCC), created by the US Department of Agriculture Economic Research Service (USDA-ERS), Washington, DC, USA to identify individuals living in rural counties if the RUCC code is 4 or greater [32,40]. Further definition and explanation of each covariate is listed in Supplementary Table S2.

### 2.5. Statistical Analysis

For both cohorts, baseline characteristics were compared between the various groups using median and inter-quartile ranges (IQRs) for continuous variables and frequency and percentages for categorical variables. The Chi-square test for categorical variables and the Mann–Whitney U test for continuous non-normal variables were used to statistically compare the data distribution between the various categories at baseline. In our primary analysis of both cohorts, after checking the proportional hazard assumptions using the Schoenfeld residuals, we deployed Cox proportional hazards models to estimate the all-cause mortality, and findings were presented as adjusted hazard ratios (aHRs). Also, competing risk modeling, using the Fine and Gray methods, was developed to calculate the subdistribution hazard ratios (sHRs) for CVE, CVm, and PCsm [41]. When a model violated the proportional hazard assumption, extra models were created after assigning the violated variables as time-varying covariates (TVCs) (Supplementary Tables S3 and S4). In these models, we used a priori set of variables to adjust for, including age, race, marital status, SES, education, hypertension, hyperlipidemia, CKD, prostate cancer grade, prostate cancer stage, surgery, and previous radiation treatment. Testing for interaction using race/ethnicity, those equal to or above the age of 75, and rurality was conducted to test for potential effect modification, and further stratification was conducted if the test was statistically significant.

A further subgroup analysis was conducted for NHB individuals, those living in low SES areas, and those living in low education areas to explore potential variations within specific subgroups. Finally, a two-year sensitivity analysis was conducted to capture early CVE in the context of typical two year ADT exposure duration and known accelerated vascular risk associated with diabetes aligning with prior literature and strategies as used by Tang et al. in patients with PC and DM [12,42,43,44,45]. All analyses were conducted using STATA/IC 16.1 (StataCorp, College Station, TX, USA), and results were deemed significant with a two-sided *p*-value of ≤0.05 [46].

## 3. Results

### 3.1. Cohort 1

We included 150,647 individuals with PC (median age 72 years, 78% NHW, 11% NHB), of whom ~32% were diabetic at baseline Figure S1. Diabetic patients were slightly older [73 (69–78) vs. 71 (68–76) years] and were more likely to identify as NHB (13% vs. 9%) or Hispanic (9% vs. 6%) compared to individuals without DM. Patients with DM were also more likely to live in areas with low SES (46% vs. 41%) and exhibit higher education levels of high school or above (59% vs. 54%). Individuals with DM were more likely to have PC grade of 3+ (45% vs. 43%) and other co-morbidities: hypertension (94% vs. 61%), hyperlipidemia (91% vs. 59%), and CKD (39% vs. 16%) (Table 1).

**Figure 1:**
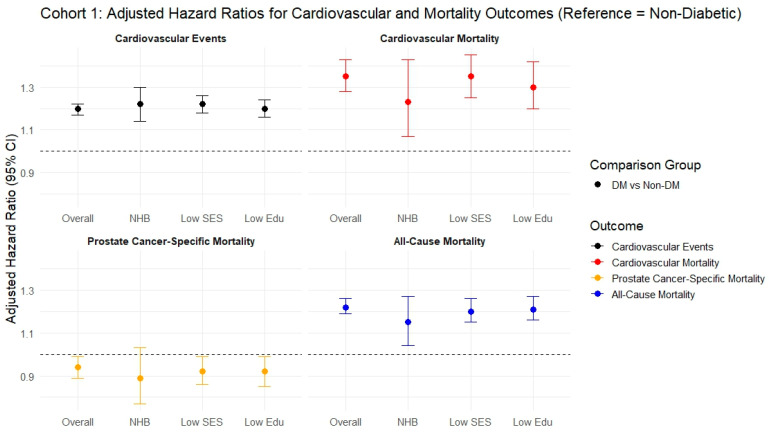
Forest plot for Fine-Gray analysis (competing risk models) and Cox regression for the various outcomes in cohorts 1 using the fully adjusted model.

Individuals were followed for a median of 3.2 years (IQR 1.46–5.83 years). During this period, individuals with DM had a significantly higher CVE rate (60% vs. 40%, *p* < 0.001), mainly attributed to heart failure (35% vs. 18%) and atrial fibrillation (29% vs. 20%), compared to individuals without DM (Table 1). The same finding was observed upon modeling in terms of CVE (sHR 1.20, 95% CI 1.17–1.22, *p* < 0.001), CVm (sHR 1.35, 95% CI 1.28–1.43, *p* < 0.001), and all-cause mortality (sHR 1.22, 95% CI 1.19–1.26, *p* < 0.001), but not with regard to PCsm (sHR 0.94, 95% CI 0.89–0.99, *p* = 0.012) (Figure 1 and Table 2). A similar pattern, with a similar effect, was noted for all three outcomes (CVE, CVm, and all-cause mortality) in the subgroup analysis of NHB, low SES, and low education (Supplementary Tables S10–S12).

Figure 1 shows adjusted hazard ratios (sHRs/aHRs) for cardiovascular and mortality outcomes in Cohort 1, comparing patients with diabetes to those without diabetes. Results are stratified by the overall population and key subgroups: NHB, low SES, and low educational attainment. Outcomes are color-coded as follows: black for cardiovascular events, red for cardiovascular mortality, orange for prostate cancer–specific mortality, and blue for all-cause mortality. The dashed horizontal line at 1.0 indicates the null value (no difference in risk). All models are adjusted for age, race/ethnicity, marital status, SES (Yost index), education, prostate cancer grade and stage, hypertension, hyperlipidemia, chronic kidney disease, surgical treatment, and radiation therapy.

When evaluating CVE, a significant interaction was demonstrated between DM and race (*p* < 0.001) in the whole sample, with a higher risk of CVE in Hispanic (sHR 1.35, 95% CI 1.25–1.46, *p* < 0.001) and NHB (sHR 1.25, 95% CI 1.18–1.33, *p* < 0.001) compared to NHW with DM (sHR 1.17, 95% CI 1.14–1.20, *p* < 0.001). A significant interaction was also found between DM and age ≥ 75 years old (*p* < 0.001), with slightly higher risk for those below 75 years (sHR 1.28, 95% CI 1.24–1.32, *p* < 0.001) compared to those above 75 years old (sHR 1.10, 95% CI 1.07–1.14, *p* < 0.001) (Table 3). Again, a similar pattern was noted in the subgroups of low SES and low education, except a significant interaction between DM and rurality (*p* = 0.011) was noted in the subgroup cohort living in low SES areas, where people with DM living in low SES areas and rural areas had slightly lower CVE (sHR 1.13, 95% CI 1.06–1.21, *p* < 0.001) compared to those living in urban areas (sHR 1.25, 95% CI 1.21–1.30, *p* < 0.001). When testing for interaction between CVm and all-cause mortality, a significant interaction was noted with those age 75 or above, where those below 75 had a higher risk of CVm and all-cause mortality. Similar findings were noted in the subgroups of low SES and low education (Supplementary Tables S5–S7).

Since some models violated the proportional hazards assumption, we modeled the relationship between diabetes and the various outcomes with diabetes as a time-varying covariate (Supplementary Tables S3 and S4). We demonstrated a similar pattern where diabetic individuals had higher risk for CVE (sHR 1.35, 95% CI 1.31–1.40, *p* < 0.001), CVm (sHR 1.55, 1.43–1.67, *p* < 0.001), and PCsm (sHR) in the overall population. We also demonstrated that the difference in impact between the DM groups and the impact on CVE decreases significantly over time when considered DM as TVC (sHR 0.96, 95% CI 0.95–0.97, *p* < 0.001) indicating that the hazard ratio between DM and CVE decreases multiplicatively over time. A similar pattern was noted in subgroup analysis and in other outcomes (Supplementary Tables S3 and S4).

After running the sensitivity analysis for two years after the beginning of the follow-up, similar findings were observed in the primary analysis and subgroup analysis (Supplementary Table S8), as well as in the interaction testing (Supplementary Table S9).

### 3.2. Cohort 2

We included 14,938 individuals with PC (median age 73 years, 79% NHW, 10% NHB, 6% Hispanic), of which 81% were individuals without DM, 16% were individuals with DM on metformin alone or metformin-based combination therapy, and 3% were individuals with DM on other medications (Table 4). Compared to individuals with DM on other medications, individuals with DM on metformin were slightly younger [72 (69–77) vs. 74 (70–79) years] and were less likely to self-identify as NHB (12% vs. 18%). Individuals with DM on metformin also were more likely to live in areas with high SES (46% vs. 39%) but less likely to live in areas with higher education levels of high school or above (54% vs. 63%) compared to those on other medications. Last, individuals with DM on metformin were more likely to have a PC grade of 3+ (66.3% vs. 64%) but less likely to have hypertension (88% vs. 92%) and CKD (35% vs. 61%), compared to individuals with DM on other medications (Table 4). Compared to Cohort 1 at baseline, participants in Cohort 2 (Table 4) differed from Cohort 1 (Table 1) by having a smaller size (*n* = 14,938 vs. *n* = 150,647), slightly older age (median 73 vs. 72 years), and a higher burden of comorbidities, including hypertension, CKD, and prior CVE. Both cohorts were predominantly Non-Hispanic White (~78–79%), with similar distributions of low SES (~42–43%) and urban residence (>82%). Rates of high-grade disease were comparable (~64%), though surgery use was lower in Cohort 2. Incident cardiovascular event rates were higher in Cohort 2 (53.8%) compared to Cohort 1 (46.6%).

During the follow-up period of ~2.5 years (IQR 1.17–4.65 years), individuals with DM on metformin had a significantly lower CVE rate (59% vs. 73%, *p* < 0.001), and reduced rates of selected outcomes [PAD (19% vs. 25%), AF (27% vs. 38%), MI (19% vs. 26%), IS (16% vs. 20%), and HF (34% vs. 50%)] compared to individuals with DM on other medications (Table 2). Upon modeling, we demonstrated higher all-cause mortality in individuals with DM on other medications compared to those on metformin (aHR 1.33, 95% CI 1.11–1.25, *p* = 0.002). This finding was further demonstrated in the subgroup analysis of NHB (aHR 2.05, 95% CI 1.29–3.27, *p* = 0.002) and in those living in low education level counties (aHR 1.71, 95% CI 1.28–2.30, *p* < 0.001) (Table 2). However, no statistically significant difference was noted between the two groups in terms of CVE, CVm, or PCsm except for PCsm in patients living in low-education counties, where individuals with DM on other medications had a significantly higher risk for PCsm than those on metformin (aHR 1.93, 95% CI 1.35–2.75, *p* < 0.0061) (Table 2). In individuals without DM on ADT, including across all subgroups, absence of diabetes was associated with a significantly lower rate of CVE (aHR 0.83, 95% CI 0.77–0.90, *p* < 0.001). (Supplementary Tables S13–S15).

When evaluating the interaction testing, while a significant interaction was demonstrated between DM and age > 75 years (*p* = 0.001) and rurality (*p* = 0.002) in the whole sample, no significant change in the direction of the relationship or the magnitude was observed (Table 3). Similar findings were demonstrated with regard to CVm; no significant interactions were noted in PCsm or all-cause mortality in the overall population and the various subgroups (Table 3 and Supplementary Tables S5–S7).

After running the sensitivity analysis for two years of follow-up, similar findings were observed in the primary analysis, subgroup analysis, and interaction testing (Supplementary Tables S8 and S9). However, the association between other DM medications and higher all-cause mortality lost its statistical significance except in low education counties, where individuals with DM on other medications demonstrated a higher risk of PCsm (sHR 2.39, 95% CI 1.54–3.71, *p* < 0.001) and all-cause mortality (aHR 2.17, 95% CI 1.34–3.53, *p* = 0.002), compared to those on metformin (Supplementary Tables S8 and S9).

**Figure 2:**
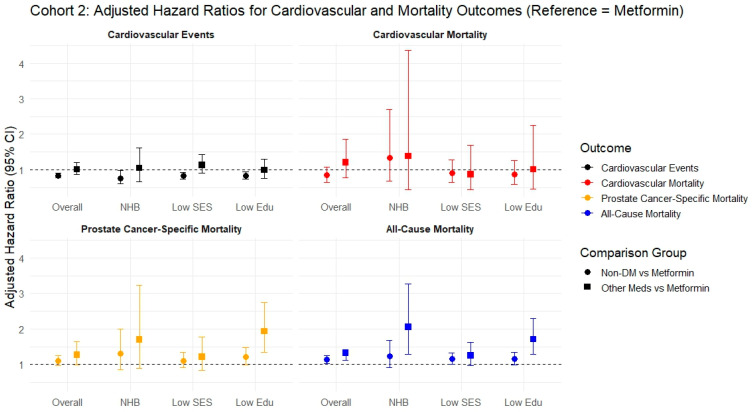
Forest plot for Fine-Gray analysis (competing risk models) and Cox regression for the various outcomes in cohorts 2 using the fully adjusted model.

Figure 2 shows adjusted hazard ratios (sHR/aHR) for cardiovascular and mortality outcomes in Cohort 2. Comparisons are made using metformin-treated patients as the ref-erence group. Two comparisons are shown: (1) non-diabetic patients vs. metformin-treated patients (circles), and (2) diabetic patients on other antidiabetic medications vs. metfor-min-treated patients (squares). Outcomes are stratified by overall population and key sub-groups: NHB, low SES, and low educational attainment. Outcomes are color-coded as follows: black for cardiovascular events, red for cardiovascular mortality, orange for prostate cancer–specific mortality, and blue for all-cause mortality. The dashed horizontal line at 1.0 indicates the null value (no difference in risk). Models are adjusted for age, race/ethnicity, marital status, SES (Yost index), educational attainment, prostate cancer grade and stage, hypertension, hyperlipidemia, chronic kidney disease, surgical treatment, and radiation therapy.

## 4. Discussion

This study examined the relationship between DM and CVD in PC survivors, as well as the impact of different DM medications in patients receiving ADT. We also evaluated these associations in high-risk subgroups, including NHB men and those residing in low-education or low SES neighborhoods. Our findings demonstrate that diabetic PC survivors faced significantly higher risks of CV events, CV mortality, and all-cause mortality compared with their non-diabetic counterparts, with similar patterns observed among NHB patients and those from disadvantaged areas. In the second cohort, PC patients receiving ADT who were treated with DM medications other than metformin had higher all-cause mortality than those taking metformin, and this association persisted even when follow-up was limited to two years.

To our knowledge, few population-based studies have quantified non-fatal CVE specifically among diabetic prostate cancer (PC) survivors, independent of ADT exposure, as most prior work emphasized mortality endpoints or ADT cohorts [47]. Our analysis adds this missing piece by showing 20–35% higher level of cardiovascular events in diabetic versus nondiabetic PC survivors, demonstrating post-diagnosis morbidity from non-fatal CVE that presents opportunities for intervention within cancer care [10]. This extends a mortality-centric literature in which diabetes is linked to higher all-cause mortality (meta-analyses) and non-PC mortality in PC survivors, with inconsistent or null effects on PC-specific mortality (PCSM) a pattern we also observed [2,3,48]. This important discussion was missed in studies assessing or comparing long term PC outcomes in DM patients [49]. Finally, our findings dovetail with growing evidence of care gaps in cardiometabolic management after a PC diagnosis where diabetes control often worsens with ADT, and recommended monitoring of lipids/HbA1c is frequently underperformed. This underscores a persistent need for integrated cardio-oncology pathways [10,13,50], particularly in non-Hispanic Black (NHB) individuals and those from low socioeconomic status (SES) and low education (Low Edu) areas in our cohort 1, who face limited healthcare access and higher CVD susceptibility, leading to worsened outcomes [31,32,51,52,53].

The elevated cardiovascular risk in prostate cancer (PC) patients with diabetes arises not only from lapses in cardiometabolic care after cancer diagnosis but also from diabetes-driven tumor–host interactions that exacerbate cardiovascular dysfunction. Lin et al. demonstrated a 10% increase in predicted 5-year CVD risk among men with type 2 DM and PC—independent of GnRH agonist use—compared with diabetic men without PC, while Griffiths et al. reported a higher, though nonsignificant, risk of acute myocardial infarction in those with both conditions relative to DM alone suggesting an additive effect of prostate tumor and PC population [47,54]. Several mechanisms underlie this effect even in non-ADT groups: shared risk factors such as obesity, hyperlipidemia, CKD and metabolic syndrome that amplify systemic inflammation [55,56,57]; increased insulin resistance in PC, which is associated with higher levels of pro-inflammatory mediators (e.g., dermcidin), impaired nitric oxide activity, and endothelial dysfunction [58]; altered pro-thrombotic adipokine profiles [55,57]; and worsened thrombo-inflammatory processes within periprostatic adipose tissue that may interact directly with the prostate tumor to further drive cardiovascular disease progression [59].

Given the high cardiovascular morbidity and mortality observed in men receiving ADT, identifying therapeutic strategies that confer both cardiometabolic and anticancer benefit is a clinical priority [60]. In our study, we observed no difference in CV events, CV mortality, or prostate cancer–specific mortality (PCSM) despite a substantial all-cause mortality benefit. This also contrasts with speculation that metformin users may have a more favorable baseline survival profile compared with other medication users [61]. In fact, a Finnish registry study reported increased PCSM among men treated with metformin, whereas other diabetes medications were not associated with such risk compared to non-users [62]. Recent high-quality evidence from the STAMPEDE randomized phase III trial found that adding metformin (850 mg twice daily) to the standard-of-care in non-diabetic men with metastatic hormone-sensitive PC did not improve overall survival, though it did attenuate ADT-associated metabolic toxicity [28,63]. The American Association of Clinical Endocrinology (AACE) acknowledges that metformin may modestly reduce cancer incidence and mortality, while its well-established cardiovascular safety in diabetes may plausibly extend to patients with cancer [64]. However, randomized evidence specifically linking metformin to reductions in major adverse cardiovascular events (MACE) or CV mortality in PC remains absent [65,66,67,68,69,70]. Although several meta-analyses and large cohorts suggest improved overall, cancer-specific, and recurrence-free survival with metformin use in PC patients, these studies rarely report incident cardiovascular outcomes [30,61,71,72,73]. Evidence comparing metformin with newer antidiabetic agents is sparse in the PC setting. GLP-1 receptor agonists and SGLT2 inhibitors, both of which provide robust cardiometabolic protection, have only limited clinical and preclinical evaluation in PC. Early reports suggest potential synergy between metformin and statins or GLP-1 analogs for anticancer effects, but outcome-level evidence is lacking [74,75,76,77].

Mechanistic data continues to support a possible dual benefit. Metformin, both alone and in combination with bicalutamide, has demonstrated immunomodulatory effects, particularly within NK and T cell subsets [78]. Its insulin-lowering action may indirectly limit tumor growth given the role of insulin and IGF-1 in PC development and progression. Additionally, metformin activates AMPK, which inhibits the mTOR pathway and related signaling cascades, thereby reducing cell proliferation. These mechanistic insights, coupled with its availability and favorable safety profile, support metformin as a candidate for therapeutic repurposing in PC. Dedicated mechanistic and outcomes-based clinical trials are warranted to define its role more clearly [79,80].

This study has several strengths, especially the comprehensive overview and the competing risk based analysis of the relationship between DM and CV outcomes in PC patients while considering the role of ADT, DM medications, and the various SDOHs. However, this study has several limitations. First, newer DM medications, including Glucagon-like peptide-1 (GLP-1) agonists and Sodium-glucose cotransporter-2 inhibitors (SGLT2i), were not included in our comparison, as they were not widely available during the study period, which ended in 2017. While these therapies have since been shown to affect cardiovascular outcomes, their use was limited at the time by the specific Food and Drug Administration (FDA) approvals [44]. Furthermore, although Hendryx et al. utilized the 2022 SEER-Medicare linkage incorporating newer treatment eras, our study was based on the 2020 linkage [81]. Mechanistically, it is also important to note that canagliflozin—the first SGLT2i evaluated—had not demonstrated any clear cardiovascular preventive benefits at that time [82]. Second, merging data from various sources could help cover any missing data gaps, but it carries some risk of underestimating or overestimating the variables used and selection bias from exclusion. Third, the cohort is restricted to patients aged 66 years and older, potentially introducing selection bias and leading to higher event rates in our analysis. Importantly, PC is predominantly a disease of older age with approximately 63% cases being diagnosed after age 65 [83]; thus, our findings are largely applicable to this age group and further studies in younger populations are warranted. Fourth, the study’s observational nature does not allow for causal inference. Fifth, residual confounding could not be excluded even after accounting for many variables that might be related to the development of CVE, CVm, PCsm, or all-cause mortality. Patients only taking metformin might have more ideal glycemic status or shorter DM duration compared to those other medications. Although our grouping strategy aligns with real-world clinical practice and prior literature, the pharmacological heterogeneity within the non-metformin part in both groups may attenuate the specificity of observed associations [35,36]. Sixth, we could only use SDOH at the census-tract or county levels due to data limitations. Seventh, due to the low sensitivity of radiation treatment data from SEER, it cannot be definitively said that a patient did not receive radiotherapy, so “no” and “unknown” categories are combined. Non-Hispanic Blacks (NHBs), those from lower socioeconomic status (SES) areas and lower education area subgroups experienced comparable to worse cardiovascular and mortality outcomes. Eighth, although the median duration of follow-up of 3.2 years captured substantial changes in outcomes, it may not have been long enough to fully assess the long term cardioprotective effects of anti-diabetic medications. CVE and mortality often occur early following prostate cancer diagnosis and initiation of ADT. Prior studies suggest that the first two years are critical in capturing these risks. Ninth, a U.S. cohort from SEER-Medicare, may not be fully generalizable to other countries due to differences in population age structures, ethnicity classifications, healthcare access, and screening patterns [84].

Future cardiovascular and oncological prostate cancer studies should aim to use longer follow ups with more individualized and granular SDOH data and compare their findings with ours. Also, other DM medications should be included, especially newer agents such as GLP-1 agonists and SGLT2 inhibitors.

## 5. Conclusions

In this large population-based SEER–Medicare analysis, diabetes was associated with significantly higher risks of cardiovascular events, cardiovascular mortality, and all-cause mortality among older men with prostate cancer, with the greatest burden observed in non-Hispanic Black patients and those from socioeconomically disadvantaged or less-educated communities. While metformin use did not reduce cardiovascular or prostate cancer–specific mortality in patients on ADT, it was significantly associated with lower all-cause mortality including high-risk subgroups, underscoring its potential as a cardiometabolically favorable option in this population.

These findings highlight two key gaps: the persistent gaps in cardio-oncologic care, especially among socially disadvantaged populations, and the lack of dedicated cardioprotective therapies for prostate cancer survivors with diabetes, particularly those receiving ADT. While our results support the continued use of metformin as part of comprehensive risk-factor management in patients on ADT, they also call for prospective studies to test whether newer diabetes agents with proven cardiovascular benefit (e.g., GLP-1 receptor agonists, SGLT2 inhibitors) can reduce the disproportionate cardiovascular burden in this high-risk group.

## Figures and Tables

**Table 1:** Baseline characteristics and cardiovascular outcomes in prostate cancer population of cohort 1 enrolled in Medicare parts A and B. The bold font represents statistically significant results.

	Total	Non-Diabetic	Diabetic	*p*-Value *
**Baseline Characteristics**				
**Sample size**	150,647	102,271 (68%)	48,376 (32%)	
**Age at cancer diagnosis, median (IQR)**	72 (68–77)	71 (68–76)	73 (69–78)	**<0.001**
**Race/ethnicity, *n* (%)**				**<0.001**
Non-Hispanic White	117,945 (78.3%)	82,924 (81.1%)	35,021 (72.4%)	
Non-Hispanic Black	16,001 (10.6%)	9523 (9.3%)	6478 (13.4%)	
Hispanic	9931 (6.6%)	5792 (5.7%)	4139 (8.6%)	
Other	6770 (4.5%)	4032 (3.9%)	2738 (5.7%)	
**Marital status, *n* (%)**				**<0.001**
Single (Never married)	8753 (5.8%)	5935 (5.8%)	2818 (5.8%)	
Married (including common law)	71,950 (47.8%)	49,973 (48.9%)	21,977 (45.4%)	
Other ^#^	14,135 (9.4%)	9152 (8.9%)	4983 (10.3%)	
Unknown	55,809 (37.0%)	37,211 (36.4%)	18,598 (38.4%)	
**Socioeconomic status (SES), *n* (%)**				**<0.001**
High SES	76,844 (51.0%)	53,509 (52.3%)	23,335 (48.2%)	
Low SES	64,398 (42.7%)	42,219 (41.3%)	22,179 (45.8%)	
Unknown	9405 (6.2%)	6543 (6.4%)	2862 (5.9%)	
**Education status, *n* (%)**				**<0.001**
High school and above	84,086 (55.8%)	55,615 (54.4%)	28,471 (58.9%)	
Below high school	66,561 (44.2%)	46,656 (45.6%)	19,905 (41.1%)	
**Rurality, *n* (%)**				**<0.001**
Rural	20,568 (13.7%)	14,617 (14.3%)	5951 (12.3%)	
Urban	130,054 (86.3%)	87,637 (85.7%)	42,417 (87.7%)	
**Use of surgery, *n* (%)**				**<0.001**
No surgery	76,608 (69.7%)	51,155 (68.2%)	25,453 (73.0%)	
Surgery	33,287 (30.3%)	23,875 (31.8%)	9412 (27.0%)	
**Use of radiotherapy, *n* (%)**				**<0.001**
No/Unknown radiation ^α^	68,398 (61.0%)	47,239 (61.7%)	21,159 (59.5%)	
Beam radiation	32,619 (29.1%)	21,442 (28.0%)	11,177 (31.4%)	
Implanted radiation	4961 (4.4%)	3573 (4.7%)	1388 (3.9%)	
Other	6099 (5.4%)	4279 (5.6%)	1820 (5.1%)	
**AJCC staging, *n* (%)**				**<0.001**
I	16,338 (10.8%)	11,297 (11.0%)	5041 (10.4%)	
II	37,511 (24.9%)	25,464 (24.9%)	12,047 (24.9%)	
III	5360 (3.6%)	3935 (3.8%)	1425 (2.9%)	
IV	7215 (4.8%)	4804 (4.7%)	2411 (5.0%)	
Unknown	84,223 (55.9%)	56,771 (55.5%)	27,452 (56.7%)	
**Grade, *n* (%)**				**<0.001**
1	16,709 (11.1%)	11,739 (11.5%)	4970 (10.3%)	
2	54,301 (36.0%)	37,831 (37.0%)	16,470 (34.0%)	
3	65,646 (43.6%)	43,969 (43.0%)	21,677 (44.8%)	
4	295 (0.2%)	180 (0.2%)	115 (0.2%)	
N/A	13,696 (9.1%)	8552 (8.4%)	5144 (10.6%)	
**ADT, *n* (%)**	30,618 (20.3%)	19,191 (18.8%)	11,427 (23.6%)	**<0.001**
**Past medical history at baseline**				
Hypertension, *n* (%)	108,021 (71.7%)	62,785 (61.4%)	45,236 (93.5%)	**<0.001**
Hyperlipidemia, *n* (%)	104,691 (69.5%)	60,694 (59.3%)	43,997 (90.9%)	**<0.001**
Chronic kidney disease, *n* (%)	34,860 (23.1%)	15,953 (15.6%)	18,907 (39.1%)	**<0.001**
Any prior cardiovascular event, *n* (%)	27,295 (18.1%)	14,157 (13.8%)	13,138 (27.2%)	**<0.001**
Peripheral arterial disease, *n* (%)	4112 (2.7%)	1872 (1.8%)	2240 (4.6%)	**<0.001**
Atrial fibrillation, *n* (%)	13,307 (8.8%)	7237 (7.1%)	6070 (12.6%)	**<0.001**
Myocardial infarction, *n* (%)	5692 (3.8%)	2731 (2.7%)	2961 (6.1%)	**<0.001**
Ischemic stroke, *n* (%)	5454 (3.6%)	2793 (2.7%)	2661 (5.5%)	**<0.001**
Heart failure, *n* (%)	10,726 (7.1%)	4654 (4.6%)	6072 (12.6%)	**<0.001**
**Outcomes**				
**Any cardiovascular event, *n* (%)**				**<0.001**
No	80,515 (53.4%)	60,974 (59.6%)	19,541 (40.4%)	
Yes	70,132 (46.6%)	41,297 (40.4%)	28,835 (59.6%)	
**Peripheral arterial disease, *n* (%)**	19,415 (12.9%)	10,707 (10.5%)	8708 (18.0%)	**<0.001**
**Atrial fibrillation, *n* (%)**	34,008 (22.6%)	19,995 (19.6%)	14,013 (28.9%)	**<0.001**
**Myocardial infarction, *n* (%)**	19,673 (13.1%)	10,630 (10.4%)	9043 (18.7%)	**<0.001**
**Ischemic stroke, *n* (%)**	18,264 (12.1%)	10,488 (10.3%)	7776 (16.1%)	**<0.001**
**Heart failure, *n* (%)**	35,570 (23.6%)	18,734 (18.3%)	16,836 (34.8%)	**<0.001**

ADT: Androgen deprivation therapy; AJCC: American Joint Committee on Cancer; * Chi-square test for categorical variables and Mann–Whitney U test for continuous non-normally distributed data; ^#^ Other marital status includes separated, divorced, and widowed; ^α^: Due to the low sensitivity of radiation treatment data from SEER, it cannot be definitively said that a patient did not receive radiotherapy, so “no” and “unknown” categories are combined.

**Table 2:** Survival analysis using Fine-Gray analysis (competing risk models) and Cox regression for the various cardiovascular outcomes in cohorts 1 and 2 using the fully adjusted model ^+^. The bold font represents statistically significant results.

**CVE (Competing Risk = All-Cause Mortality)** **sHR (95% CI, *p*-Value)**					
		Overall	NHB	Low SES *	Low Edu ^#^
**Cohort 1**	Event/Total, person-year ^#^	40,197/109,895 429,629.7	4242/11,769 45,676.7	18,524/48,934 186,892.5	17,682/51,878 207,193.9
	Non-DM	Reference			
	DM	**1.20 (1.17–1.22, *p* < 0.001)**	**1.22 (1.14–1.30, *p* < 0.001)**	**1.22 (1.18–1.26, *p* < 0.001)**	**1.20 (1.16–1.24, *p* < 0.001)**
**Cohort 2**	Event/Total, person-year ^#^	4036/9735 32,523.6	428/1021 3381.8	1896/4385 14,167.5	1863/4744 16,028.2
	Non-DM	**0.83 (0.77–0.90, *p* < 0.001)**	**0.76 (0.60–0.97, *p* = 0.026)**	**0.83 (0.73–0.93, *p* = 0.002)**	**0.83 (0.73–0.94, *p* = 0.003)**
	DM on metformin alone or metformin-based combination therapy (Reference)				
	DM on other medications	1.01 (0.86–1.20, *p* = 0.869)	1.04 (0.66–1.62, *p* = 0.877)	1.13 (0.90–1.42, *p* = 0.288)	0.99 (0.75–1.29, *p* = 0.916)
**CVm (Competing risk = All-cause mortality except CVD mortality)** **sHR (95% CI, *p*-value)**					
		Overall	NHB	Low SES *	Low Edu ^#^
	Non-DM	Reference			
	DM	**1.35 (1.28–1.43, *p* < 0.001)**	**1.23 (1.07–1.43, *p* = 0.004)**	**1.35 (1.25–1.45, *p* < 0.001)**	**1.30 (1.20–1.42, *p* < 0.001)**
**Cohort 2**	Event/Total, person-year ^#^	433/9735 32,523.6	57/1021 3381.8	226/4385 14,167.5	183/4744 16,028.2
	Non-DM	0.84 (0.65–1.08, *p* = 0.181)	1.34 (0.67–2.71, *p* = 0.408)	0.91 (0.65–1.28, *p* = 0.596)	0.86 (0.59–1.26, *p* = 0.448)
	DM on metformin alone or metformin-based combination therapy (Reference)				
	DM on other medications	1.20 (0.77–1.86, *p* = 0.415)	1.38 (0.43–4.36, *p* = 0.588)	0.87 (0.44–1.69, *p* = 0.675)	1.01 (0.45–2.25, *p* = 0.983)
**PCsm (Competing risk = All-cause mortality except PCsm)** **sHR (95% CI, *p*-value)**					
		Overall	NHB	Low SES *	Low Edu ^#^
	Non-DM	Reference			
	DM	**0.94 (0.89–0.99, *p* = 0.012)**	0.89 (0.77–1.03, *p* = 0.122)	**0.92 (0.86–0.99, *p* = 0.028)**	**0.92 (0.85–0.99, *p* = 0.028)**
**Cohort 2**	Event/Total, person-year ^#^	2078/9735 32,523.6	251/1021 3381.8	1001/4385 14,167.5	934/4744 16,028.2
	Non-DM	1.09 (0.96–1.25, *p* = 0.188)	1.31 (0.86–2.00, *p* =0.209)	1.10 (0.91–1.34, *p* = 0.314)	1.21 (0.98–1.48, *p* = 0.070)
	DM on metformin alone or metformin-based combination therapy Reference				
	DM on other medications	1.27 (0.99–1.64, *p* = 0.065)	1.70 (0.89–3.24, *p* = 0.107)	1.21 (0.83–1.77, *p* = 0.325)	**1.93 (1.35–2.75, *p* < 0.001)**
**All-cause mortality (Cox)** **aHR (95% CI, *p*-value)**					
		Overall	NHB	Low SES *	Low Edu ^#^
	Non-DM	Reference			
	DM	**1.22 (1.19–1.26, *p* < 0.001)**	**1.15 (1.04–1.27, *p* = 0.005)**	**1.20 (1.15–1.26, *p* < 0.001)**	**1.21 (1.16–1.27, *p* < 0.001)**
**Cohort 2**	Event/Total, person-year ^#^	3160/9734 40,838.6	399/1021 4262.2	1571/4385 18,046.4	1380/4744 19,643.2
	Non-DM	**1.13 (1.02–1.25, *p* = 0.025)**	1.23 (0.91–1.68, *p* = 0.183)	1.15 (1.00–1.33, *p* = 0.056)	1.15 (0.98–1.34, *p* = 0.083)
	DM on metformin alone or metformin-based combination therapy Reference				
	DM on other medications	**1.33 (1.11–1.25, *p* = 0.002)**	**2.05 (1.29–3.27, *p* = 0.002)**	1.25 (0.96–1.63, *p* = 0.095)	**1.71 (1.28–2.30, *p* < 0.001)**

aHR: adjusted hazard ratio; CVm: cardiovascular mortality; CVE: cardiovascular events; DM: diabetes mellitus; Edu: education; NHB: non-Hispanic Blacks; PCsm: prostate cancer-specific mortality; SES: socio-economic status; sHR: subdistribution hazard ratio; * Low SES defined as Yost Index ≤ 2; Low Education defined as high school education < 25%. ^+^ Model adjustment: Age, race, marital status, SES (Yost index), education, prostate cancer grade, prostate cancer stage, hypertension, hyperlipidemia and chronic kidney disease, surgery, and radiation use. ^#^ Total analysis time at risk and under observation, presented using person-time.

**Table 3:** Interaction testing and consequent stratification for the various cardiovascular outcomes in cohorts 1 and 2 for the overall populations ^+^. The bold font represents statistically significant results. Further stratification was only performed if interaction was found to be significant.

**Cohort 1**								
	**CVE**		**CVm**		**PCsm**		**All-Cause Mortality**	
	**sHR (95% CI, *p*-Value)**						**aHR (95% CI, *p*-Value)**	
	Interaction	Stratification	Interaction	Stratification	Interaction	Stratification	Interaction	Stratification
**Race**	***p* < 0.001**	**DM + White: 1.17 (1.14–1.20, *p* < 0.001)** **DM + Black: 1.25 (1.18–1.33, *p* < 0.001)** **DM + Hispanic: 1.35 (1.25–1.46, *p* < 0.001)** **DM + Other: 1.33 (1.20–1.46, *p* < 0.001)**	*p* = 0.543	-	*p* = 0.179	-	*p* = 0.229	-
**Age ≥ 75**	***p* < 0.001**	**DM + Age < 75: 1.28 (1.24–1.32, *p* < 0.001)** **DM + Age ≥ 75: 1.10 (1.07–1.14, *p* < 0.001)**	***p* < 0.001**	**DM + Age < 75: 1.65 (1.51–1.79, *p* < 0.001)** **DM + Age ≥ 75: 1.21 (1.13–1.29, *p* < 0.001)**	***p* = 0.002**	DM + Age < 75: 1.04 (0.96–1.12, *p* = 0.390) **DM + Age ≥ 75: 0.89 (0.84–0.95, *p* < 0.001)**	***p* < 0.001**	**DM + Age < 75: 1.33 (1.27–1.39, *p* < 0.001)** **DM + Age ≥ 75: 1.16 (1.12–1.21, *p* < 0.001)**
**Rurality**	*p* = 0.087	-	*p* = 0.091	-	*p* = 0.418		***p* = 0.001**	**DM + Rural: 1.32 (1.23–1.41, *p* < 0.001)** **DM + Urban: 1.21 (1.17–1.24, *p* < 0.001)**
**Cohort 2**								
	**CVE**		**CVm**		**PCsm**		**All-Cause Mortality**	
	**sHR (95% CI, *p*-Value)**						**aHR (95% CI, *p*-Value)**	
	Interaction	Stratification	Interaction	Stratification	Interaction	Stratification	Interaction	Stratification
**Race**	*p* = 0.765	-	***p* < 0.001**	Non-DM + White: 0.78 (0.58–1.05, *p* = 0.106) Non-DM + Black: 1.13 (0.55–2.32, *p* = 0.737) Non-DM + Hispanic: 1.06 (0.49–2.30, *p* = 0.883) Non-DM + Other: 0.87 (0.35–2.19, *p* = 0.769) DM on other + White: 1.55 (0.94–2.55, *p* = 0.084) DM on other + Black: 1.21 (0.37–3.94, *p* = 0.757) **DM on other + Hispanic: (*p* < 0.001)** ^†^ DM on other + Other: 0.57 (0.07–4.59, *p* = 0.593)	*p* = 0.725	-	*p* = 0.244	-
**Age ≥ 75**	***p* = 0.001**	**Non-DM + Age < 75: 0.86 (0.77–0.95, *p* = 0.005)** **Non-DM + Age ≥ 75: 0.79 (0.70–0.90, *p* = 0.001)** DM on other + Age < 75: 1.17 (0.93–1.48, *p* = 0.174) DM on other + Age ≥ 75: 0.86 (0.68–1.10, *p* = 0.243)	*p* = 0.216	-	*p* = 0.222	-	*p* = 0.279	-
**Rurality**	***p* = 0.002**	**Non-DM + Rural: 0.80 (0.66–0.97, *p* = 0.021)** **Non-DM + Urban: 0.84 (0.77–0.92, *p* < 0.001)** DM on other + Rural: 1.11 (0.78–1.59, *p* = 0.566) DM on other + Urban: 0.99 (0.82–1.20, *p* = 0.935)	*p* = 0.204	-	*p* = 0.143	-	*p* = 0.481	-

aHR: adjusted hazard ratio; CVm: cardiovascular mortality; CVE: cardiovascular events; DM: diabetes mellitus; PCsm: prostate cancer-specific mortality; sHR: subdistribution hazard ratio. ^†^ For some subgroup analyses, the statistical software returned extremely small hazard ratio estimates (HR < 0.001) with confidence intervals also reported as <0.001. These p values are presented exactly as obtained from the statistical output, although they should be interpreted with caution given the instability of estimates in strata with very low event counts. ^+^ Model adjustment: Age, race, marital status, SES (Yost index), education, prostate cancer grade, prostate cancer stage, hypertension, hyperlipidemia and chronic kidney disease, surgery, and radiation use.

**Table 4:** Baseline characteristics and cardiovascular outcomes in prostate cancer population of cohort 2 who received ADT and enrolled in Medicare part D. The bold font represents statistically significant results.

	Total	Non-DM	DM on Metformin Alone or Metformin-Based Combination Therapy	DM on Other Medication(s) ^^^	*p*-Value *
**Baseline Characteristics**					
**Sample size**	14,938	12,071 (80.8%)	2356 (15.8%)	511 (3.4%)	
**Age at cancer diagnosis, median (IQR)**	73 (69–78)	73 (69–78)	72 (69–77)	74 (70–79)	**<0.001**
**Race/ethnicity, *n* (%)**					**<0.001**
Non-Hispanic White	11,850 (79.3%)	9871 (81.8%)	1648 (69.9%)	331 (64.8%)	
Non-Hispanic Black	1436 (9.6%)	1069 (8.9%)	276 (11.7%)	91 (17.8%)	
Hispanic	931 (6.2%)	625 (5.2%)	251 (10.7%)	55 (10.8%)	
Other	721 (4.8%)	506 (4.2%)	181 (7.7%)	34 (6.7%)	
**Marital status, *n* (%)**					0.61
Single (Never married)	1016 (6.8%)	824 (6.8%)	157 (6.7%)	35 (6.8%)	
Married (including common law)	6468 (43.3%)	5208 (43.1%)	1048 (44.5%)	212 (41.5%)	
Other ^#^	1442 (9.7%)	1178 (9.8%)	207 (8.8%)	57 (11.2%)	
Unknown	6012 (40.2%)	4861 (40.3%)	944 (40.1%)	207 (40.5%)	
**Socioeconomic status (SES), *n* (%)**					**<0.001**
High SES	7354 (49.2%)	6076 (50.3%)	1077 (45.7%)	201 (39.3%)	
Low SES	6405 (42.9%)	5067 (42.0%)	1077 (45.7%)	261 (51.1%)	
Unknown	1179 (7.9%)	928 (7.7%)	202 (8.6%)	49 (9.6%)	
**Education status, *n* (%)**					**<0.001**
High school and above	8098 (54.2%)	6506 (53.9%)	1269 (53.9%)	323 (63.2%)	
Below high school	6840 (45.8%)	5565 (46.1%)	1087 (46.1%)	188 (36.8%)	
**Rurality, *n* (%)**					0.70
Rural	2549 (17.1%)	2072 (17.2%)	388 (16.5%)	89 (17.4%)	
Urban	12,388 (82.9%)	9998 (82.8%)	1968 (83.5%)	422 (82.6%)	
**Use of surgery, *n* (%)**					**0.015**
No surgery	7616 (78.2%)	6107 (77.9%)	1223 (78.8%)	286 (84.4%)	
Surgery	2119 (21.8%)	1737 (22.1%)	329 (21.2%)	53 (15.6%)	
**Use of radiotherapy, *n* (%)**					**0.012**
No/Unknown radiation ^α^	5313 (53.8%)	4309 (54.2%)	808 (51.1%)	196 (56.8%)	
Beam radiation	3683 (37.3%)	2914 (36.6%)	636 (40.2%)	133 (38.6%)	
Implanted radiation	188 (1.9%)	>150 (>1.50%) ^+^	26 (1.6%)	<11 ^+^	
Other	696 (7.0%)	>570 (>7.0%) ^+^	111 (7.0%)	>13 (>3.5%) ^+^	
**AJCC Staging, *n* (%)**					0.43
I	345 (2.3%)	283 (2.3%)	50 (2.1%)	12 (2.3%)	
II	2672 (17.9%)	2121 (17.6%)	452 (19.2%)	99 (19.4%)	
III	710 (4.8%)	577 (4.8%)	113 (4.8%)	20 (3.9%)	
IV	2103 (14.1%)	1709 (14.2%)	312 (13.2%)	82 (16.0%)	
Unknown	9108 (61.0%)	7381 (61.1%)	1429 (60.7%)	298 (58.3%)	
**Grade, *n* (%)**					**0.004**
1	400 (2.7%)	318 (2.6%)	66 (2.8%)	16 (3.1%)	
2	2771 (18.6%)	2245 (18.6%)	448 (19.0%)	78 (15.3%)	
3	9652 (64.6%)	>7760 (>64.0%) ^+^	>1560 (>66.0%) ^+^	>320 (>63.0%) ^+^	
4	48 (0.3%)	>40 (>0.2%) ^+^	<11 ^+^	<11 ^+^	
N/A	2067 (13.8%)	1700 (14.1%)	275 (11.7%)	92 (18.0%)	
**Past medical history at baseline**					
Hypertension, *n* (%)	11,446 (76.6%)	8903 (73.8%)	2074 (88.0%)	469 (91.8%)	**<0.001**
Hyperlipidemia, *n* (%)	10,784 (72.2%)	8359 (69.2%)	1989 (84.4%)	436 (85.3%)	**<0.001**
Chronic kidney disease, *n* (%)	4211 (28.2%)	3077 (25.5%)	821 (34.8%)	313 (61.3%)	**<0.001**
Any prior cardiovascular event, *n* (%)	3450 (23.1%)	2654 (22.0%)	594 (25.2%)	202 (39.5%)	**<0.001**
Peripheral arterial disease, *n* (%)	559 (3.7%)	411 (3.4%)	106 (4.5%)	42 (8.2%)	**<0.001**
Atrial fibrillation, *n* (%)	1695 (11.4%)	1328 (11%)	277 (11.8%)	90 (17.6%)	**<0.001**
Myocardial infarction, *n* (%)	784 (5.2%)	574 (4.8%)	153 (6.5%)	57 (11.2%)	**<0.001**
Ischemic stroke, *n* (%)	731 (4.9%)	545 (4.5%)	144 (6.1%)	42 (8.2%)	**<0.001**
Heart failure, *n* (%)	1375 (9.2%)	1011 (8.4%)	248 (10.5%)	116 (22.7%)	**<0.001**
**Outcomes**					
**Any cardiovascular event, *n* (%)**					**<0.001**
No	6901 (46.2%)	5786 (47.9%)	977 (41.5%)	138 (27.0%)	
Yes	8037 (53.8%)	6285 (52.1%)	1379 (58.5%)	373 (73.0%)	
**Peripheral arterial disease, *n* (%)**	2325 (15.6%)	1762 (14.6%)	438 (18.6%)	125 (24.5%)	**<0.001**
**Atrial fibrillation, *n* (%)**	3842 (25.7%)	3018 (25%)	632 (26.8%)	192 (37.6%)	**<0.001**
**Myocardial infarction, *n* (%)**	2337 (15.6%)	1752 (14.5%)	451 (19.1%)	134 (26.2%)	**<0.001**
**Ischemic stroke, *n* (%)**	2093 (14.0%)	1613 (13.4%)	380 (16.1%)	100 (19.6%)	**<0.001**
**Heart failure, *n* (%)**	4168 (27.9%)	3117 (25.8%)	797 (33.8%)	254 (49.7%)	**<0.001**

AJCC: American Joint Committee on Cancer; DM: diabetes; * Chi-square test for categorical variables and Mann–Whitney U test for continuous non-normally distributed data; ^#^ Other marital status includes separated, divorced, and widowed; ^^^ Other medications include alpha-glucosidase inhibitors, amylin analogs, dipeptidyl peptidase-4 (DPP-4), insulin, meglitinides, sulfonylureas, thiazolidinedione; ^α^: Due to the low sensitivity of radiation treatment data from SEER, it cannot be definitively said that a patient did not receive radiotherapy, so “no” and “unknown” categories are combined. ^+^ Cell sizes less than 11 are suppressed in accordance with cell size suppression policy in data use agreement and privacy regulations; values are reported as ranges or approximations to protect patient confidentiality.

## Data Availability

This study used data from the SEER-Medicare linked database, which contains individual-level health information and is not publicly available due to privacy and legal restrictions. Access to the SEER-Medicare data requires approval from the National Cancer Institute and the Centers for Medicare & Medicaid Services (https://healthcaredelivery.cancer.gov/seermedicare/overview/, accessed on 16 August 2024).

## Supplementary Materials

The following supporting information can be downloaded at: https://www.mdpi.com/article/10.3390/cancers17172854/s1, Figure S1: CONSORT Flow Diagram; Table S1: Description of outcomes and exposure, and their definitions; Table S2: Description of the different covariates and their definitions; Table S3: Proportional hazard and sub-distribution hazard assumption testing; Table S4: Survival analysis with time-varying covariate analysis (diabetes) using Fine-Gray analysis (competing risk models) for the various cardiovascular outcomes in relevant cohorts using fully adjusted model +. The bold font represents statistically significant results; Table S5: Interaction testing and consequent stratification for the various cardiovascular outcomes in cohorts 1 and 2 for the non-Hispanic Blacks population. + The bold font represents statistically significant results; Table S6: Interaction testing and consequent stratification for the various cardiovascular outcomes in cohorts 1 and 2 for the population living in low-socioeconomic status neighborhoods +. The bold font represents statistically significant results; Table S7: Interaction testing and consequent stratification for the various cardiovascular outcomes in cohorts 1 and 2 for the population living in low-education neighborhoods + The bold font represents statistically significant results; Table S8: Sensitivity analysis for the various cardiovascular outcomes within 2 years of follow-up in cohorts 1 and 2. The bold font represents statistically significant results; Table S9: Interaction testing for the sensitivity analysis for the various cardiovascular outcomes within 2 years of follow-up in cohorts 1 and 2. The bold font represents statistically significant results; Table S10: Baseline characteristics and cardiovascular outcomes in low education subgroup of cohort 1 and enrolled in Medicare parts A and B. The bold font represents statistically significant results; Table S11: Baseline characteristics and cardiovascular outcomes in low socio-economic status subgroup of cohort 1 and enrolled in Medicare parts A and B. The bold font represents statistically significant results; Table S12: Baseline characteristics and cardiovascular outcomes in non-Hispanic Black subgroup of cohort 1 and enrolled in Medicare parts A and B. The bold font represents statistically significant results; Table S13: Baseline characteristics and cardiovascular outcomes in low education subgroup of cohort 2 and enrolled in Medicare parts A, B and D. The bold font represents statistically significant results; Table S14: Baseline characteristics and cardiovascular outcomes in low socio-economic status subgroup of cohort 2 and enrolled in Medicare parts A, B and D. The bold font represents statistically significant results; Table S15: Baseline characteristics and cardiovascular outcomes in non-Hispanic Black subgroup of cohort 2 and enrolled in Medicare parts A, B and D. The bold font represents statistically significant results; Table S16: Baseline characteristics of the excluded patients.

## Abbreviations

The following abbreviations are used in this manuscript:

aHR	Adjusted Hazard Ratio
AF	Atrial Fibrillation
ADT	Androgen Deprivation Therapy
aOR	Adjusted Odds Ratio
BMI	Body Mass Index
CI	Confidence Interval
CKD	Chronic Kidney Disease
CVD	Cardiovascular Disease
CVE	Cardiovascular Event
CVm	Cardiovascular Mortality
DM	Diabetes Mellitus
GLP-1	Glucagon-Like Peptide-1
HbA1c	Hemoglobin A1c
HLD	Hyperlipidemia
HF	Heart Failure
HR	Hazard Ratio
HTN	Hypertension
IQR	Interquartile Range
IS	Ischemic Stroke
LDL	Low-Density Lipoprotein
MetAb-Pro	Metformin and Abiraterone Study in Prostate Cancer
NCI	National Cancer Institute
NHB	Non-Hispanic Black
NHW	Non-Hispanic White
PAD	Peripheral Artery Disease
PC	Prostate Cancer
PCsm	Prostate Cancer-Specific Mortality
SD	Standard Deviation
SDOH	Social Determinants of Health
SEER	Surveillance, Epidemiology, and End Results
SES	Socioeconomic Status
sHR	Subdistribution Hazard Ratio
SGLT2	Sodium-Glucose Cotransporter-2
