# Supplementary material for: Impact of Diabetes and Metformin on Cardiovascular Outcomes in Prostate Cancer Patients Aged 66 and Older: The Role of Social Determinants of Health and Racial Disparities
Source: Cancers (Basel). 2025 Aug 30;17(17):2854. doi: 10.3390/cancers17172854 (PMC12427577; doi:10.3390/cancers17172854)

Supplemental Tables

Definitions

Supplementary Table S1 Description of outcomes and exposure, and their definitions.

Variables	Explanation and ICD-9 and ICD-10 codes used
**Exposures**	
**Diabetes mellitus**	• 250.xx: Diabetes and related complications<br>• 362.0x: Diabetes retinopathy<br>• chronic condition flag files
**Diabetes medications**	• Metformin users- metformin alone, or in combination of other diabetes medications<br>• Other users- alpha-glucosidase inhibitors, amylin analogs, dipeptidyl peptidase-4 (DPP-4), insulin, meglitinides, sulfonylureas, thiazolidinedione
**Outcomes**	○
**Heart failure**	• 428.XX: Congestive heart failure (unspecified).<br>• 402.01: Hypertensive heart disease with heart failure, benign.<br>• 402.11: Hypertensive heart disease with heart failure, malignant.<br>• 402.91: Hypertensive heart disease with heart failure, unspecified.<br>• 414.8: Other specified forms of chronic ischemic heart disease.<br>• I50.XX: Heart failure (acute, chronic, unspecified).<br>• I11.00: Hypertensive heart disease with heart failure.<br>• I13.0/I13.2: Hypertensive heart and chronic kidney disease with heart failure.<br>• I42.X/I43.XX: Cardiomyopathies.
**Ischemic stroke**	• 431.XX: Intracerebral hemorrhage.<br>• 435.0X–435.9X: Transient cerebral ischemia.<br>• 433.01–433.91: Occlusion and stenosis of precerebral arteries with or without infarction.

	• 434.01–434.91: Occlusion of cerebral arteries with or without infarction.<br>• 997.01: Iatrogenic cerebrovascular infarction or hemorrhage.<br>• 344.60/344.61: Hemiplegia due to cerebrovascular disease.<br>• I63/I66: Cerebral infarction and arterial occlusion.<br>• G45X: Transient ischemic attacks.<br>• G834: Monoplegia.<br>• G9781: Cerebrovascular complications.
**Acute myocardial infarction**	• 411.XX: Unstable angina and related conditions.<br>• 410.XX: Acute myocardial infarction (STEMI/NSTEMI).<br>• I20.0: Unstable angina.<br>• I21.X: Acute myocardial infarction.<br>• I24.X: Other acute ischemic heart diseases.
**Atrial fibrillation**	• 427.31: Atrial fibrillation (unspecified type).<br>• I48.91: Atrial fibrillation, unspecified.
**Peripheral artery disease**	• 440.2×: Atherosclerosis of native arteries of the extremities.<br>• 440.3×: Atherosclerosis of bypass graft of extremities.<br>• 440.8×: Other forms of atherosclerosis.<br>• 440.9×: Generalized and unspecified atherosclerosis.<br>• 443.9×: Peripheral vascular disease, unspecified.<br>• I70.0: Atherosclerosis of the aorta.<br>• I70.2: Atherosclerosis of native arteries of the extremities.<br>• I73.9: Peripheral vascular disease, unspecified.

ICD: International classification of diseases.

Supplementary Table S2 Description of the different covariates and their definitions.

Variables	Explanation
**Covariates**	
**Age at diagnosis**	Study includes only patients aged 65 years and above.
**Race**	Recoded field with classification as:<br>• White (reference)<br>• Black<br>• Other
**Ethnicity**	Hispanic; non-Hispanic
**Marital status**	Marital Status has the following categories:<br>• Unmarried-Single (reference)<br>• Married
**Socioeconomic Yost Index**	Measuring socioeconomic status across geographic areas
**Rurality**	Rurality status (yes vs no) was defined according to 2013 Rural-Urban Continuum Codes (RUCC).<br><br>It is a categorization system that differentiates metropolitan counties based on the population size of their metropolitan area and classifies nonmetropolitan counties according to their level of urbanization and proximity to a metropolitan area<br><br>Positive rural status: patients residing in areas with a population of <2,500 or <20,000 not adjacent to metropolitan area (according to RUCC codes 7,8, or 9)
**County level educational status**	Percentage of people >25 years old with less than a high school diploma and percentage of people >25 years old with only a high school diploma
**CVD**	Composite at any time after PC diagnosis:<br>• Heart Failure: (HF)<br>• Atrial Fibrillation: (AF)<br>• Acute Myocardial Infarction: (AMI)<br>• Peripheral Artery Disease: (PAD)<br>Ischemic Stroke: (IS)
**Chronic Kidney Disease**	Classified as:<br>• 0 (reference; no history of diagnosis or diagnosis after prostate cancer diagnosis)

	1 (history of diagnosis before the prostate cancer diagnosis)
**Hypertension**	Classified as:<br>• 0 (reference; no history of diagnosis or diagnosis after prostate cancer diagnosis)<br>• 1 (history of diagnosis before the prostate cancer diagnosis)
**Hyperlipidemia**	Classified as:<br>• 0 (reference; no history of diagnosis or diagnosis after prostate cancer diagnosis)<br>• 1 (history of diagnosis before the prostate cancer diagnosis)
**AJCC TNM Stage**	American Joint Committee on Cancer (AJCC) stage at prostate cancer diagnosis categorized as:<br>• 1 (reference)<br>• 2 (includes II, II not otherwise specified, IIA, IIB, IIC)<br>• 3 (includes III, III not otherwise specified, IIIA, IIIB, IIIC)<br>• 4 (includes IV, IV not otherwise specified, IVA, IVB)
**Histology**	
**Tumor Grade**	Grade at prostate cancer diagnosis categorized as:<br>• 1 (reference)<br>• 2<br>• 3<br>• 4
**Androgen Deprivation Therapy**	Classified as:<br>• 0 (did not receive leuprolide ADT)<br>• 1 (received leuprolide ADT)
**Specific Androgen Deprivation Therapy Medications**	Apalutaminde, Bicalutamide, Darolutamide, Degarelix acetate, Enzalutamide, Flutamide, Goserelin acetate, Leuprolide acetate
**Chemotherapy**	Classified as:<br>• 0 (did not receive chemotherapy)<br>• 1 (received chemotherapy)
**Radiotherapy**	Classified as:<br>• 0 (did not receive radiotherapy)<br>• Beam radiation<br>• Implanted radiation
**Surgery**	Classified as:<br>• 0 (reference)

- Radical prostatectomy
- Laparoscopic prostatectomy
- Bilateral orchiectomy
- TURP

ADT: Androgen deprivation therapy; CVD: cardiovascular disease; TURP: transurethral resection of the prostate.

Hazard Testing

Supplementary Table S3 Proportional hazard and subdistribution hazard assumption testing.

	Cohort 1	Cohort 2	
CVE	**P<0.001**	P=0.955	
CVm	**P=0.002**	P=0.138	
PCsm	**P<0.001**	**P=0.045**	
All-cause mortality	P=0.5488	P=0.6242	

Supplementary Table S4 Survival analysis with time-varying covariate analysis (diabetes) using Fine-Gray analysis (competing risk models) for the various cardiovascular outcomes in relevant cohorts using fully adjusted model.+ The bold font represents statistically significant results.

		Overall	NHB	Low SES*	Low Edu[#]
**CVE (Competing risk = All-cause mortality) sHR (95% CI, p-value)**					
**Cohort 1**	Non-DM	Reference			
	DM	**1.35 (1.31-1.40, P<0.001) TVC: 0.96 (0.95-0.97, p<0.001)**	**1.34 (1.22-1.48, p<0.001) TVC: 0.97 (0.95-0.99, p=0.005)**	**1.33 (1.27-1.39, p<0.001) TVC: 0.97 (0.96-0.98, p<0.001)**	**1.39 (1.33-1.46, p<0.001) TVC: 0.95 (0.94-0.96, p<0.001)**
**CVm (Competing risk = All-cause mortality except CVD mortality) sHR (95% CI, p-value)**					
**Cohort 1**	Non-DM	Reference			
	DM	**1.55 (1.43-1.67, p<0.001) TVC:0.94 (0.92-0.97, p<0.001)**	**1.47 (1.20-1.82, p<0.001) TVC:0.93 (0.87-0.99, p=0.018)**	**1.50 (1.35-1.66, p<0.001) TVC: 0.96 (0.92-0.99, p=0.007)**	**1.45 (1.29-1.63, p<0.001) TVC: 0.96 (0.92-0.99, p=0.014)**
**PCsm (Competing risk = All-cause mortality except PCsm) sHR (95% CI, p-value)**					
**Cohort 1**	Non-DM	Reference			
	DM	**1.17 (1.10-1.24, p=0.003) TVC: 0.90 (0.88-0.92, p<0.001)**	1.13 (0.94-1.34, p=0.187) **TVC: 0.88 (0.83-0.94, p<0.001)**	**1.11 (1.02-1.21, p=0.016) TVC: 0.90 (0.87-0.93, p<0.001)**	**1.15 (1.04-1.26, p=0.005) TVC: 0.89 (0.86-0.92, p<0.001)**
**Cohort 2**	Non-DM	0.97 (0.80-1.18, p=0.795) TVC: 1.05 (0.99-1.12, p=0.129)	1.04 (0.51-2.12, p=0.907) TVC: 1.10 (0.86-1.41, p=0.448)	0.88 (0.67-1.16, p=0.359) **TVC: 1.11 (1.00-1.22, p=0.045)**	1.13 (0.83-1.54, p=0.434) TVC: 1.03 (0.94-1.13, p=0.578)
	DM on metformin	Reference			
	DM on other medications	**1.70 (1.22-2.37, p=0.002)**	2.40 (0.97-5.91, p=0.058)	**1.63 (1.01-2.63, p=0.046)**	**2.07 (1.31-3.25, p=0.002)**

	**TVC: 0.86 (0.76-0.98, p=0.025)**	TVC: 0.82 (0.58-1.16, p=0.266)	TVC: 0.84 (0.68-1.03, p=0.093)	TVC: 0.96 (0.83-1.12, p=0.636)

CVm: cardiovascular mortality; CVE: cardiovascular events; DM: diabetes mellitus; Edu: education; NHB: non-Hispanic Blacks; PCsm: prostate cancer-specific mortality; SES: socio-economic status; sHR: subdistribution hazard ratio; *Low SES defined as Yost Index ≤ 2; Low Education defined as high school education <25%. +Model adjustment: Age, race, marital status, SES (Yost index), education, prostate cancer grade, prostate cancer stage, hypertension, hyperlipidemia and chronic kidney disease, surgery, and radiation use.

Interaction Testing in Subgroups

Supplementary Table S5 Interaction testing and consequent stratification for the various cardiovascular outcomes in cohorts 1 and 2 for the non-Hispanic Blacks population. +The bold font represents statistically significant results.

**Cohort 1**								
	**CVE**		**CVm**		**PCsm**		**All-cause Mortality**	
	**sHR (95% CI, p-value)**						**aHR (95% CI, p-value)**	
	Interaction	Stratification	Interaction	Stratification	Interaction	Stratification	Interaction	Stratification
**Race**	-	-	-	-	-	-	-	-
**Age ≥75**	P=0.486	**DM + Age <75: 1.28 (1.24-1.32, P<0.001)** **DM + Age ≥75: 1.10 (1.07-1.14, P<0.001)**	P=0.496	-	P=0.792	-	P=0.454	-
**Rurality**	P=0.691	-	P=0.666	-	P=0.067	-	P=0.273	-
**Cohort 2**								
	**CVE**		**CVm**		**PCsm**		**All-cause mortality**	
	**sHR (95% CI, p-value)**						**aHR (95% CI, p-value)**	
	Interaction	Stratification	Interaction	Stratification	Interaction	Stratification	Interaction	Stratification
**Race**	-	-	-	-	-	-	-	-
**Age ≥75**	P=0.128	**-**	**P<0.001**	Non-DM + Age <75: 0.84 (0.40-1.77, P=0.651) **Non-DM + Age ≥75: 8.87e+06 (5.42e+06- 1.45e+07, P<0.001)** DM on other + Age <75: 1.73 (0.54-5.55, P=0.359) DM on other + Age ≥75: 0.71 (0.40-1.26, P=0.247)	P=0.663	-	P=0.365	-
**Rurality**	P=0.031	**Non-DM + Rural: 0.47 (0.25-0.90, P=0.022)** Non-DM + Urban: 0.80 (0.62-1.04, P=0.096) DM on other + Rural: 2.00 (0.62-6.50, P=0.247) DM on other + Urban: 0.97 (0.60-1.57, P=0.914)	**P<0.001**	**Non-DM + Rural: 5.33e+06 (1.53e+06- 1.86e+07, P<0.001)** Non-DM + Urban: 1.10 (0.52-2.31, P=0.803) **DM on other + Rural: 6.36e+06 (601925- 6.72e+07, P<0.001)** DM on other + Urban: 1.06 (0.30-3.77, P=0.928)	P=0.449	-	P=0.385	-

aHR: adjusted hazard ratio; CVm: cardiovascular mortality; CVE: cardiovascular events; DM: diabetes mellitus; PCsm: prostate cancer-specific mortality; sHR: subdistribution hazard ratio

$^{+}$Model adjustment: Age, race, marital status, SES (Yost index), education, prostate cancer grade, prostate cancer stage, hypertension, hyperlipidemia and chronic kidney disease, surgery, and radiation use.

Supplementary Table S6 Interaction testing and consequent stratification for the various cardiovascular outcomes in cohorts 1 and 2 for the population living in low-socioeconomic status neighborhoods+. The bold font represents statistically significant results.

**Cohort 1**								
	**CVE**		**CVm**		**PCsm**		**All-cause Mortality**	
	**sHR (95% CI, p-value)**						**aHR (95% CI, p-value)**	
	Interaction	Stratification	Interaction	Stratification	Interaction	Stratification	Interaction	Stratification
**Race**	**P=0.004**	**DM + White: 1.18 (1.13-1.22, P<0.001)** **DM + Black: 1.29 (1.20-1.38, P<0.001)** **DM + Hispanic: 1.36 (1.24-1.50, P<0.001)** **DM + Other: 1.34 (1.16-1.55, P<0.001)**	P=0.752	-	**P=0.014**	DM + White: 0.99 (0.90-1.07, P=0.734) **DM + Black: 0.80 (0.69-0.93, P=0.005)**	P=0.142	-
**Age ≥75**	**P=0.001**	**DM + Age <75: 1.28 (1.23-1.33, P<0.001)** **DM + Age ≥75: 1.15 (1.10-1.21, P<0.001)**	**P<0.001**	**DM + Age <75: 1.62 (1.44-1.81, P<0.001)** **DM + Age ≥75: 1.20 (1.09-1.32, P=0.001)**	**P=0.012**	DM + Age <75: 1.03 (0.92-1.14, P=0.607) **DM + Age ≥75: 0.87 (0.79-0.95, P=0.002)**	**P<0.001**	**DM + Age <75: 1.32 (1.23-1.40, P<0.001)** **DM + Age ≥75: 1.13 (1.07-1.20, P<0.001)**
**Rurality**	**P=0.008**	**DM + Rural: 1.13 (1.06-1.21, P<0.001)** **DM + Urban: 1.25 (1.21-1.30, P<0.001)**	P=0.783	-	P=0.067	P=0.051	**P=0.029**	**DM + Rural: 1.33 (1.22-1.45, P<0.001)** **DM + Urban: 1.18 (1.12-1.23, P<0.001)**
**Cohort 2**								
	**CVE**		**CVm**		**PCsm**		**All-cause mortality**	
	**sHR (95% CI, p-value)**						**aHR (95% CI, p-value)**	
	Interaction	Stratification	Interaction	Stratification	Interaction	Stratification	Interaction	Stratification
**Race**	P=0.613	-	**P<0.001**	Non-DM + White: 0.96 (0.59-1.54, P=0.851) Non-DM + Black: 1.08 (0.51-2.30, P=0.844)	P=0.332	-	P=0.074	-

				Non-DM + Hispanic: 0.79 (0.35-1.79, P=0.568)<br>Non-DM + Other: 0.69 (0.20-2.33, P=0.547)<br>DM on other + White: 1.30 (0.55-3.07, P=0.556)<br>DM on other + Black: 1.02 (0.28-3.76, P=0.978)<br>**DM on other + Hispanic: <0.001 (<0.001-<0.001, P<0.001)**<br>DM on other + Other: 1.12 (0.13-9.61, P=0.917)				
**Age ≥75**	**P=0.001**	Non-DM + Age <75: 0.92 (0.79-1.07, P=0.282)<br>**Non-DM + Age ≥75: 0.71 (0.59-0.86, P<0.001)**<br>DM on other + Age <75: 1.33 (0.99-1.78, P=0.060)<br>DM on other + Age ≥75: 0.92 (0.65-1.30, P=0.636)	P=0.564	-	P=0.494	-	P=0.439	-
**Rurality**	**P=0.004**	Non-DM + Rural: 0.87 (0.68-1.11, P=0.250)<br>**Non-DM + Urban: 0.82 (0.72-0.94, P=0.004)**<br>DM on other + Rural: 1.40 (0.87-2.25, P=0.165)	P=0.496	**-**	P=0.669	-	P=0.907	-

	DM on other + Urban: 1.08 (0.84-1.40, P=0.557)			

aHR: adjusted hazard ratio; CVm: cardiovascular mortality; CVE: cardiovascular events; DM: diabetes mellitus; PCsm: prostate cancer-specific mortality; sHR: subdistribution hazard ratio

$^{+}$Model adjustment: Age, race, marital status, SES (Yost index), education, prostate cancer grade, prostate cancer stage, hypertension, hyperlipidemia and chronic kidney disease, surgery, and radiation use.

Supplementary Table S7 Interaction testing and consequent stratification for the various cardiovascular outcomes in cohorts 1 and 2 for the population living in low-education neighborhoods. +The bold font represents statistically significant results.

	CVE		CVm		PCsm		All-cause Mortality	
**Cohort 1**								
	**CVE**		**CVm**		**PCsm**		**All-cause Mortality**	
	**sHR (95% CI, p-value)**						**aHR (95% CI, p-value)**	
	Interaction	Stratification	Interaction	Stratification	Interaction	Stratification	Interaction	Stratification
**Race**	P=0.114	-	P=0.954	-	P=0.637	-	P=0.135	-
**Age ≥75**	**P<0.001**	**DM + Age <75: 1.26 (1.21-1.32, P<0.001) DM + Age ≥75: 1.12 (1.07-1.18, P<0.001)**	**P<0.001**	**DM + Age <75: 1.73 (1.52-1.98, P<0.001) DM + Age ≥75: 1.13 (1.02-1.25, P=0.019)**	P=0.225	-	**P=0.017**	**DM + Age <75: 1.31 (1.21-1.41, P<0.001) DM + Age ≥75: 1.17 (1.10-1.24, P<0.001)**
**Rurality**	P= 0.726	-	P=0.184	-	P=0.278	-	P=0.151	-
**Cohort 2**								
	**CVE**		**CVm**		**PCsm**		**All-cause mortality**	
	**sHR (95% CI, p-value)**						**aHR (95% CI, p-value)**	
	Interaction	Stratification	Interaction	Stratification	Interaction	Stratification	Interaction	Stratification
**Race**	P=0.771	-	**P<0.001**	Non-DM + White: 1.02 (0.62-1.67, P=0.942) Non-DM + Black: 0.64 (0.20-2.05, P=0.457) Non-DM + Hispanic: 0.83 (0.33-2.10, P=0.698) Non-DM + Other: 0.52 (0.15-1.82, P=0.303) DM on other + White: 2.08 (0.84-5.13, P=0.112) DM on other + Black: 0.62 (0.06-6.20, P=0.687) **DM on other + Hispanic: <0.001 (<0.001-<0.001, P<0.001)**	P=0.675	-	P=0.732	-

				**DM on other + Other: <0.001 (<0.001-<0.001, P<0.001)**				
**Age ≥75**	**P=0.015**	Non-DM + Age <75: 0.90 (0.76-1.05, P=0.177)<br>**Non-DM + Age ≥75: 0.76 (0.63-0.91, P=0.004)**<br>DM on other + Age <75: 1.29 (0.91-1.84, P=0.156)<br>DM on other + Age ≥75: 0.75 (0.51-1.12, P=0.162)	P=0.754	-	**P=0.003**	Non-DM + Age <75: 1.18 (0.91-1.53, P=0.223)<br>Non-DM + Age ≥75: 1.24 (0.91- 1.68, =0.171)<br>**DM on other + Age <75: 2.37 (1.53-3.67, P<0.001)**<br>DM on other + Age ≥75: 1.54 (0.88-2.70, P=0.130)	P=0.404	-
**Rurality**	P=0.077	-	P=0.376	**-**	**P<0.001**	Non-DM + Rural: 1.38 (0.57-3.33, P=0.472)<br>Non-DM + Urban: 1.20 (0.98-1.48, P=0.084)<br>**DM on other + Rural: <0.001 (<0.001 -<0.001, P<0.001)**<br>**DM on other + Urban: 1.97 (1.39-2.81, P<0.001)**	P=0.863	-

aHR: adjusted hazard ratio; CVm: cardiovascular mortality; CVE: cardiovascular events; DM: diabetes mellitus; PCsm: prostate cancer-specific mortality; sHR: subdistribution hazard ratio

+Model adjustment: Age, race, marital status, SES (Yost index), education, prostate cancer grade, prostate cancer stage, hypertension, hyperlipidemia and chronic kidney disease, surgery, and radiation use.

Sensitivity analysis

Supplementary Table S8 Sensitivity analysis for the various cardiovascular outcomes within 2 years of follow-up in cohorts 1 and 2. The bold font represents statistically significant results.

sHR (95% CI)		Overall	NHB	Low SES* (Yost Index ≤ 2)	Low Edu* (High school education <25%)
**CVE (Competing risk = All-cause mortality) sHR (95% CI, p-value)**					
**Cohort 1**	Non-DM	Reference			
	DM	**1.20 (1.16-1.24, P<0.001)**	**1.19 (1.08-1.32, P=0.001)**	**1.20 (1.14-1.26, P<0.001)**	**1.20 (1.14-1.26, P<0.001)**
**Cohort 2**	Non-DM	**0.82 (0.73-0.92, P=0.001)**	0.73 (0.52-1.05, P=0.086)	**0.76 (0.65-0.89, P=0.001)**	0.89 (0.75-1.06, P=0.192)
	DM on Metformin	Reference			
	DM on other medications	0.96 (0.76-1.20, P=0.703)	1.18 (0.65-2.14, P=0.587)	0.97 (0.72-1.31, P=0.839)	1.02 (0.71-1.49, P=0.902)
**CVm (Competing risk = All-cause mortality except CVD mortality) sHR (95% CI, p-value)**					
**Cohort 1**	Non-DM	Reference			
	DM	**1.35 (1.26-1.45, P<0.001)**	**1.22 (1.00-1.50, P=0.050)**	**1.35 (1.22-1.49, P<0.001)**	**1.26 (1.12-1.41, P<0.001)**
**Cohort 2**	Non-DM	0.95 (0.66-1.235, P=0.755)	1.58 (0.56-4.49, P=0.389)	1.17 (0.71-1.92, P=0.546)	1.36 (0.75-2.44, P=0.306)
	DM on Metformin	Reference			
	DM on other medications	0.89 (0.45-1.76, P=0.740)	1.50 (0.26-8.78, P=0.651)	0.66 (0.22-1.97, P=0.458)	1.60 (0.55-4.67, P=0.391)
**PCsm (Competing risk = All-cause mortality except PCsm) sHR (95% CI, p-value)**					
**Cohort 1**	Non-DM	Reference			
	DM	0.94 (0.89-1.00, P=0.060)	0.91 (0.77-1.08, P=0.289)	0.92 (0.85-1.00, P=0.061)	0.94 (0.85-1.03, P=0.171)

**Cohort 2**	Non-DM	1.12 (0.93-1.34, P=0.252)	0.97 (0.56-1.68, p =0.925)	1.00 (0.78-1.28, P=0.999)	**1.45 (1.07-1.98, P=0.017)**
	DM on Metformin	Reference			
	DM on other medications	**1.40 (1.02-1.92, P=0.036)**	1.43 (0.64-3.19, P=0.378)	1.14 (0.72-1.82, P=0.574)	**2.39 (1.54-3.71, P<0.001)**
**All-cause mortality (Cox) aHR (95% CI, p-value)**					
**Cohort 1**	Non-DM	Reference			
	DM	**1.12 (1.07-1.17, P<0.001)**	1.06 (0.93-1.20, P=0.390)	**1.12 (1.05-1.19, P<0.001)**	**1.11 (1.04-1.19, P=0.003)**
**Cohort 2**	Non-DM	**1.20 (1.00-1.43, P=0.050)**	1.13 (0.68-1.89, P=0.643)	1.13 (0.89-1.44, P=0.320)	**1.55 (1.16-2.07, P=0.003)**
	DM on Metformin	Reference			
	DM on other medications	0.96 (0.69-1.34, P=0.813)	1.47 (0.68-3.18, P=0.327)	0.89 (0.56-1.42, P=0.626)	**2.17 (1.34-3.53, P=0.002)**

aHR: adjusted hazard ratio; CVm: cardiovascular mortality; CVE: cardiovascular events; DM: diabetes mellitus; Edu: education; NHB: non-Hispanic Blacks; PCsm: prostate cancer-specific mortality; SES: socio-economic status; sHR: subdistribution hazard ratio; *Low SES defined as Yost Index ≤ 2; Low Education defined as high school education <25%.

Model adjustment: Age, race, marital status, SES (Yost index), education, prostate cancer grade, prostate cancer stage, hypertension, hyperlipidemia and chronic kidney disease, surgery, and radiation use.

Supplementary Table S9 Interaction testing for the sensitivity analysis for the various cardiovascular outcomes within 2 years of follow-up in cohorts 1 and 2. The bold font represents statistically significant results.

**Overall Population**								
**Cohort 1**								
	**CVE**		**CVm**		**PCsm**		**All-cause Mortality**	
	**sHR (95% CI, p-value)**						**aHR (95% CI, p-value)**	
	Interaction	Stratification	Interaction	Stratification	Interaction	Stratification	Interaction	Stratification
**Race**	P=0.246	-	P=0.895	-	P=0.205	-	P=0.200	-
**Age ≥75**	**P<0.001**	**DM + Age <75: 1.30 (1.24-1.36, P<0.001) DM + Age ≥75: 1.11 (1.06-1.15, P<0.001)**	**P<0.001**	**DM + Age <75: 1.70 (1.51-1.91, P<0.001) DM + Age ≥75: 1.21 (1.11-1.31, P<0.001)**	P=0.051	-	**P<0.001**	**DM + Age <75: 1.27 (1.18-1.37, P<0.001) DM + Age ≥75: 1.07 (1.02-1.13, P=0.010)**
**Rurality**	P=0.124	-	P=0.113	-	P=0.811		P=0.122	-
**Cohort 2**								
	**CVE**		**CVm**		**PCsm**		**All-cause mortality**	
	**sHR (95% CI, p-value)**						**aHR (95% CI, p-value)**	
	Interaction	Stratification	Interaction	Stratification	Interaction	Stratification	Interaction	Stratification
**Race**	P=0.643	-	**P<0.001**	Non-DM + White: 0.81 (0.54-1.22, P=0.320) Non-DM + Black: 1.43 (0.50-4.14, P=0.507) Non-DM + Hispanic: 1.16 (0.41-3.30, P=0.779) Non-DM + Other: 2.70 (0.34-21.47, P=0.348) DM on other + White: 1.05 (0.49-2.25, P=0.900) DM on other + Black: 1.27 (0.23-7.16, P=0.783) **DM on other + Hispanic: <0.001**	P=0.342	-	P=0.435	-

				**(<0.001-<0.001, P<0.001)**<br>**DM on other + Other: <0.001 (<0.001-<0.001, P<0.001)**				
**Age ≥75**	**P=0.012**	Non-DM + Age <75: 0.90 (0.77-1.06, P=0.198)<br>**Non-DM + Age ≥75: 0.72 (0.61-0.85, P<0.001)**<br>DM on other + Age <75: 1.19 (0.87-1.61, P=0.279)<br>DM on other + Age ≥75: 0.74 (0.53-1.04, P=0.086)	P=0.863	-	P=0.232	-	P=0.359	-
**Rurality**	**P=0.018**	Non-DM + Rural: 0.83 (0.64-1.08, P=0.159)<br>**Non-DM + Urban: 0.81 (0.71-0.92, P=0.002)**<br>DM on other + Rural: 1.06 (0.66-1.70, P=0.820)<br>DM on other + Urban: 0.93 (0.72-1.21, P=0.583)	P=0.968	-	P=0.106	-	**P=0.023**	Non-DM + Rural: 1.23 (0.84-1.81, P=0.288)<br>Non-DM + Urban: 1.19 (0.97-1.45, P=0.096)<br>**DM on other + Rural: 0.34 (0.12-0.98, P=0.046)**<br>DM on other + Urban: 1.16 (0.81-1.66, P=0.416)
**Non-Hispanic Black Population**								
**Cohort 1**								
	**CVE**		**CVm**		**PCsm**		**All-cause Mortality**	
	**sHR (95% CI, p-value)**						**aHR (95% CI, p-value)**	
	Interaction	Stratification	Interaction	Stratification	Interaction	Stratification	Interaction	Stratification
**Race**	-	-	-	-	-	-	**-**	-
**Age ≥75**	P=0.417	-	P=0.981	-	P=0.687	-	P=0.421	-
**Rurality**	P=0.357	-	P=0.543	-	P=0.619		P=0.863	-
**Cohort 2**								

	CVE		CVm		PCsm		All-cause mortality	
	**sHR (95% CI, p-value)**						**aHR (95% CI, p-value)**	
	Interaction	Stratification	Interaction	Stratification	Interaction	Stratification	Interaction	Stratification
**Race**	-	-	-	-	-	-	-	-
**Age ≥75**	P=0.154	-	Not converging	-	P=0.898	-	P=0.834	-
**Rurality**	**P=0.017**	Non-DM + Rural: 0.51 (0.11-2.34, P=0.389)<br>Non-DM + Urban: 0.72 (0.50-1.04, P=0.079)<br>**DM on other + Rural: 6.09 (1.11-33.41, P=0.037)**<br>DM on other + Urban: 0.96 (0.51-1.81, P=0.894)	**P<0.001**	**Non-DM + Rural: 1.83e+06 (407413.22-8.24e+06, P<0.001)**<br>Non-DM + Urban: 1.38 (0.49-3.91, P=0.542)<br>**DM on other + Rural: 5.16e+06 (664715.42-4.00e+07, P<0.001)**<br>DM on other + Urban: 0.81 (0.08-8.00, P=0.855)	P=0.187	-	P=0.101	-
**Low Socio-Economic Population**								
**Cohort 1**								
	**CVE**		**CVm**		**PCsm**		**All-cause Mortality**	
	**sHR (95% CI, p-value)**						**aHR (95% CI, p-value)**	
	Interaction	Stratification	Interaction	Stratification	Interaction	Stratification	Interaction	Stratification
**Race**	P=0.098	**DM + White: 1.17 (1.14-1.20, P<0.001)**<br>**DM + Black: 1.25 (1.18-1.33, P<0.001)**<br>**DM + Hispanic: 1.35 (1.25-1.46, P<0.001)**<br>**DM + Other: 1.33 (1.20-1.46, P<0.001)**	P=0.955	-	**P=0.045**	DM + White: 0.99 (0.89-1.10, P=0.864)<br>**DM + Black: 0.82 (0.69-0.99, P=0.034)**<br>DM + Hispanic: 0.87 (0.69-1.09, P=0.228)<br>**DM + Other: 0.67 (0.49-0.91, P=0.012)**	**P=0.032**	**DM + White: 1.18 (1.10-1.27, P<0.001)**<br>DM + Black: 1.04 (0.92-1.18, P=0.535)<br>DM + Hispanic: 1.01 (0.84-1.21, P=0.937)<br>DM + Other: 0.87 (0.68-1.13, P=0.298)
**Age ≥75**	**P=0.014**	**DM + Age <75: 1.27 (1.19-1.35, P<0.001)**	**P=0.004**	**DM + Age <75: 1.62 (1.38-1.90, P<0.001)**	P=0.152	-	**P=0.040**	**DM + Age <75: 1.24 (1.12-1.37, P<0.001)**

	CVE Interaction	CVE Stratification	CVm Interaction	CVm Stratification	PCsm Interaction	PCsm Stratification	All-cause mortality Interaction	All-cause mortality Stratification
		**DM + Age ≥75: 1.13 (1.06-1.21, P<0.001)**		**DM + Age ≥75: 1.22 (1.08-1.38, P=0.001)**				DM + Age ≥75: 1.07 (0.99-1.15, P=0.079)
**Rurality**	P=0.084	-	P=0.651	-	P=0.817	-	P=0.616	-
**Cohort 2**								
	**CVE**		**CVm**		**PCsm**		**All-cause mortality**	
	**sHR (95% CI, p-value)**						**aHR (95% CI, p-value)**	
	Interaction	Stratification	Interaction	Stratification	Interaction	Stratification	Interaction	Stratification
**Race**	P=0.517	-	P<0.001	Non-DM + White: 1.27 (0.62-2.58, P=0.511) Non-DM + Black: 1.16 (0.39-3.44, P=0.783) Non-DM + Hispanic: 0.84 (0.27-2.58, P=0.756) Non-DM + Other: 1.95 (0.23-16.93, P=0.544) DM on other + White: 1.16 (0.31-4.33, P=0.821) DM on other + Black: 0.65 (0.07-5.97, P=0.707) **DM on other + Hispanic: <0.001 (<0.001-<0.001, P<0.001)** **DM on other + Other: <0.001 (<0.001-<0.001, P<0.001)**	P=0.335	-	P=0.098	-
**Age ≥75**	**P=0.007**	Non-DM + Age <75: 0.84 (0.68-1.04, P=0.116) **Non-DM + Age ≥75: 0.67 (0.52-0.84, P=0.001)** DM on other + Age <75: 1.00 (0.67-1.52, P=0.983)	P=0.691	-	P=0.667	-	P=0.578	-

		DM on other + Age ≥75: 0.91 (0.59-1.41, P=0.672)						
**Rurality**	**P=0.005**	Non-DM + Rural: 0.97 (0.69-1.36, P=0.859) **Non-DM + Urban: 0.70 (0.59-0.85, P<0.001)** DM on other + Rural: 1.39 (0.77-2.50, P=0.278) DM on other + Urban: 0.87 (0.61-1.24, P=0.442)	P=0.773	-	P=0.919	-	P=0.385	-
**Low-Education Population**								
**Cohort 1**								
	**CVE**		**CVm**		**PCsm**		**All-cause Mortality**	
	**sHR (95% CI, p-value)**						**aHR (95% CI, p-value)**	
	Interaction	Stratification	Interaction	Stratification	Interaction	Stratification	Interaction	Stratification
**Race**	P=0.668	-	P=0.956	-	P=0.821	-	P=0.380	-
**Age ≥75**	**P=0.002**	**DM + Age <75: 1.30 (1.21-1.39, P<0.001)** **DM + Age ≥75: 1.12 (1.05-1.19, P=0.001)**	**P<0.001**	**DM + Age <75: 1.72 (1.42-2.08, P<0.001)** DM + Age ≥75: 1.10 (0.96-1.25, P=0.166)	P=0.391	-	**P=0.025**	**DM + Age <75: 1.28 (1.13-1.45, P<0.001)** DM + Age ≥75: 1.05 (0.98-1.14, P=0.183)
**Rurality**	P= 0.928	-	P= 0.397	-	P=0.164	-	**P=0.028**	**DM + Rural: 1.50 (1.04-2.16, P=0.028)** **DM + Urban: 1.10 (1.03-1.18, P=0.006)**
**Cohort 2**								
	**CVE**		**CVm**		**PCsm**		**All-cause mortality**	
	**sHR (95% CI, p-value)**						**aHR (95% CI, p-value)**	
	Interaction	Stratification	Interaction	Stratification	Interaction	Stratification	Interaction	Stratification

**Race**	P=0.468	-	P<0.001	Non-DM + White: 1.48 (0.71-3.10, P=0.294) Non-DM + Black: 0.92 (0.18-4.60, P=0.915) Non-DM + Hispanic: 1.01 (0.24-4.23, P=0.987) **Non-DM + Other: 1.83e+06 (568218.34-5.91e+06, P<0.001)** DM on other + White: 2.61 (0.76-9.00, P=0.130) DM on other + Black: 1.28 (0.10-15.93, P=0.846) **DM on other + Hispanic: <0.001 (<0.001-<0.001, P<0.001)** DM on other + Other: 0.59 (0.27-1.29, P=0.187)	P=0.963	-	P=0.467	-
**Age ≥75**	P=0.349	-	P=0.871	-	**P=0.001**	**Non-DM + Age <75: 1.60 (1.02-2.51, P=0.041)** Non-DM + Age ≥75: 1.33 (0.88-2.02, P=0.176) **DM on other + Age <75: 3.16 (1.78-5.63, P<0.001)** DM on other + Age ≥75: 1.77 (0.91-3.44, P=0.090)	P=0.281	-
**Rurality**	P=0.633	-	Not converging	-	Not converging	-	P=0.286	-

aHR: adjusted hazard ratio; CVm: cardiovascular mortality; CVE: cardiovascular events; DM: diabetes mellitus; Edu: education; NHB: non-Hispanic Blacks; PCsm: prostate cancer-specific mortality; SES: socio-economic status; sHR: subdistribution hazard ratio; *Low SES defined as Yost Index ≤ 2; Low Education defined as high school education <25%.
Model adjustment: Age, race, marital status, SES (Yost index), education, prostate cancer grade, prostate cancer stage, hypertension, hyperlipidemia and chronic kidney disease, surgery, and radiation use.

Baseline Tables of Subgroups

Supplementary Table S10 Baseline characteristics and cardiovascular outcomes in low education subgroup of cohort 1 and enrolled in Medicare parts A and B. The bold font represents statistically significant results.

Low Education+	Total	Non-Diabetic	Diabetic	P-value*
**Baseline Characteristics**				
**Sample size**	66,561	46,656	19,905	
**Age at cancer diagnosis (median, IQR)**	72 (68-77)	71 (68-76)	74 (70-79)	**<0.001**
**Race/Ethnicity, n (%)**				**<0.001**
**Non-Hispanic White**	51,425 (77.3%)	37,479 (80.3%)	13,946 (70.1%)	
**Non-Hispanic Black**	5,797 (8.7%)	3,640 (7.8%)	2,157 (10.8%)	
**Hispanic**	5,384 (8.1%)	3,162 (6.8%)	2,222 (11.2%)	
**Other**	3,955 (5.9%)	2,375 (5.1%)	1,580 (7.9%)	
**Marital Status, n (%)**				**<0.001**
**Single (Never married)**	4,337 (6.5%)	3,056 (6.6%)	1,281 (6.4%)	
**Married (Including common law)**	34,352 (51.6%)	24,620 (52.8%)	9,732 (48.9%)	
**Other**	6,130 (9.2%)	4,040 (8.7%)	2,090 (10.5%)	
**Unknown**	21,742 (32.7%)	14,940 (32.0%)	6,802 (34.2%)	
**Socioeconomic Status (SES), n (%)**				**<0.001**
**High SES**	41,238 (62.0%)	29,502 (63.2%)	11,736 (59.0%)	
**Low SES**	23,323 (35.0%)	15,667 (33.6%)	7,656 (38.5%)	

**Unknown**	2,000 (3.0%)	1,487 (3.2%)	513 (2.6%)	
**Patient Rurality, n (%)**				**<0.001**
**Rural**	1,672 (2.5%)	1,314 (2.8%)	358 (1.8%)	
**Urban**	64,874 (97.5%)	45,330 (97.2%)	19,544 (98.2%)	
**Prostate cancer stage, n (%)**				**<0.001**
**I**	7,556 (11.4%)	5,439 (11.7%)	2,117 (10.6%)	
**II**	17,119 (25.7%)	12,086 (25.9%)	5,033 (25.3%)	
**III**	2,719 (4.1%)	2,047 (4.4%)	672 (3.4%)	
**IV**	3,434 (5.2%)	2,322 (5.0%)	1,112 (5.6%)	
**Unknown**	35,733 (53.7%)	24,762 (53.1%)	10,971 (55.1%)	
**Grade, n (%)**				**<0.001**
**1**	7,441 (11.2%)	5,440 (11.7%)	2,001 (10.1%)	
**2**	24,056 (36.1%)	17,279 (37.0%)	6,777 (34.0%)	
**3**	29,006 (43.6%)	20,101 (43.1%)	8,905 (44.7%)	
**4**	122 (0.2%)	73 (0.2%)	49 (0.2%)	
**9**	5,936 (8.9%)	3,763 (8.1%)	2,173 (10.9%)	
**Surgery, n (%)**				**<0.001**
**No Surgery**	35,486 (68.4%)	24,497 (67.0%)	10,989 (71.8%)	
**Surgery**	16,392 (31.6%)	12,085 (33.0%)	4,307 (28.2%)	
**Radiotherapy, n (%)**				**<0.001**
**No/Unknown Radiotherapy**[α]	32,630 (62.2%)	23,236 (62.8%)	9,394 (60.7%)	
**Beam Radiation**	14,302 (27.2%)	9,719 (26.3%)	4,583 (29.6%)	
**Implanted Radiation**	2,421 (4.6%)	1,786 (4.8%)	635 (4.1%)	
**Other**	3,137 (6.0%)	2,269 (6.1%)	868 (5.6%)	
**Chemotherapy, n (%)**	650 (1.2%)	477 (1.3%)	173 (1.1%)	0.11
**ADT, n (%)**	13,900 (20.9%)	8,986 (19.3%)	4,914 (24.7%)	**<0.001**
**Past medical history at baseline**				
**Hypertension, n (%)**	45,431 (68.3%)	27,081 (58.0%)	18,350 (92.2%)	**<0.001**
**Hyperlipidemia, n (%)**	45,323 (68.1%)	27,186 (58.3%)	18,137 (91.1%)	**<0.001**
**Chronic Kidney Disease, n (%)**	14,540 (21.8%)	6,843 (14.7%)	7,697 (38.7%)	**<0.001**
**CEV Prior, n (%)**	11,039 (16.6%)	5,994 (12.8%)	5,045 (25.3%)	**<0.001**
**PAD Prior, n (%)**	1,507 (2.3%)	726 (1.6%)	781 (3.9%)	**<0.001**

**Atrial Fibrillation Prior, n (%)**	5,716 (8.6%)	3,263 (7.0%)	2,453 (12.3%)	**<0.001**
**Myocardial Infarction Prior, n (%)**	2,123 (3.2%)	1,072 (2.3%)	1,051 (5.3%)	**<0.001**
**Ischemic Stroke Prior, n (%)**	2,124 (3.2%)	1,127 (2.4%)	997 (5.0%)	**<0.001**
**Heart Failure Prior, n (%)**	4,057 (6.1%)	1,833 (3.9%)	2,224 (11.2%)	**<0.001**
**Outcomes**				
**CEV, n (%)**				**<0.001**
**No**	37,479 (56.3%)	28,954 (62.1%)	8,525 (42.8%)	
**Yes**	29,082 (43.7%)	17,702 (37.9%)	11,380 (57.2%)	
**Had PAD in Claims, n (%)**	7,752 (11.6%)	4,493 (9.6%)	3,259 (16.4%)	**<0.001**
**Had Atrial Fibrillation in Claims, n (%)**	14,471 (21.8%)	8,838 (18.9%)	5,633 (28.3%)	**<0.001**
**Had MI in Claims, n (%)**	7,836 (11.8%)	4,354 (9.3%)	3,482 (17.5%)	**<0.001**
**Had Ischemic Stroke in Claims, n (%)**	7,464 (11.2%)	4,452 (9.5%)	3,012 (15.1%)	**<0.001**
**Had HF in Claims, n (%)**	14,011 (21.0%)	7,589 (16.3%)	6,422 (32.3%)	**<0.001**

[+]Low Education defined as high school education <25%. ADT: Androgen deprivation therapy; AJCC: American Joint Committee on Cancer; *Chi-square test for categorical variables and Mann-Whitney U test for continuous non-normally distributed data; [#]Other marital status includes separated, divorced, and widowed; α**:** Due to the low sensitivity of radiation treatment data from SEER, it cannot be definitively said that a patient did not receive radiotherapy, so "no" and "unknown" categories are combined

Supplementary Table S11 Baseline characteristics and cardiovascular outcomes in low socio-economic status subgroup of cohort 1 and enrolled in Medicare parts A and B. The bold font represents statistically significant results.

**Low Socio-Economic Status[+]**	**Total**	**Non-Diabetic**	**Diabetic**	**P-value***
**Baseline Characteristics**				
**Sample size**	64,398	42,219	22,179	
**Age at cancer diagnosis (median, IQR)**	72 (68-77)	71 (68-76)	73 (69-78)	**<0.001**
**Race/Ethnicity, n (%)**				**<0.001**
**Non-Hispanic White**	43,547 (67.6%)	30,119 (71.3%)	13,428 (60.5%)	
**Non-Hispanic Black**	11,452 (17.8%)	6,761 (16.0%)	4,691 (21.2%)	
**Hispanic**	6,461 (10.0%)	3,650 (8.6%)	2,811 (12.7%)	

**Other**	2,938 (4.6%)	1,689 (4.0%)	1,249 (5.6%)	
**Marital Status, n (%)**				**<0.001**
**Single (Never married)**	5,052 (7.8%)	3,250 (7.7%)	1,802 (8.1%)	
**Married (Including common law)**	29,121 (45.2%)	19,486 (46.2%)	9,635 (43.4%)	
**Other**	7,339 (11.4%)	4,721 (11.2%)	2,618 (11.8%)	
**Unknown**	22,886 (35.5%)	14,762 (35.0%)	8,124 (36.6%)	
**Education, n (%)**				**<0.001**
**High Education**	41,075 (63.8%)	26,552 (62.9%)	14,523 (65.5%)	
**Low Education**	23,323 (36.2%)	15,667 (37.1%)	7,656 (34.5%)	
**Patient Rurality, n (%)**				**<0.001**
**Rural**	13,003 (20.2%)	9,208 (21.8%)	3,795 (17.1%)	
**Urban**	51,395 (79.8%)	33,011 (78.2%)	18,384 (82.9%)	
**Prostate cancer stage, n (%)**				**<0.001**
**I**	7,227 (11.2%)	4,761 (11.3%)	2,466 (11.1%)	
**II**	16,985 (26.4%)	11,175 (26.5%)	5,810 (26.2%)	
**III**	2,231 (3.5%)	1,587 (3.8%)	644 (2.9%)	
**IV**	3,562 (5.5%)	2,349 (5.6%)	1,213 (5.5%)	
**Unknown**	34,393 (53.4%)	22,347 (52.9%)	12,046 (54.3%)	
**Grade, n (%)**				**<0.001**
**1**	6,696 (10.4%)	4,535 (10.7%)	2,161 (9.7%)	
**2**	22,492 (34.9%)	14,990 (35.5%)	7,502 (33.8%)	
**3**	28,542 (44.3%)	18,602 (44.1%)	9,940 (44.8%)	
**4**	143 (0.2%)	85 (0.2%)	58 (0.3%)	
**9**	6,525 (10.1%)	4,007 (9.5%)	2,518 (11.4%)	
**Surgery, n (%)**				**<0.001**
**No Surgery**	34,817 (71.2%)	22,483 (69.9%)	12,334 (73.6%)	
**Surgery**	14,117 (28.8%)	9,696 (30.1%)	4,421 (26.4%)	
**Radiotherapy, n (%)**				**<0.001**
**No/Unknown Radiotherapy**[α]	30,726 (61.9%)	20,364 (62.3%)	10,362 (61.0%)	
**Beam Radiation**	14,101 (28.4%)	8,957 (27.4%)	5,144 (30.3%)	
**Implanted Radiation**	2,130 (4.3%)	1,495 (4.6%)	635 (3.7%)	
**Other**	2,702 (5.4%)	1,863 (5.7%)	839 (4.9%)	

**Chemotherapy, n (%)**	535 (1.1%)	380 (1.2%)	155 (0.9%)	**0.010**
**ADT, n (%)**	13,699 (21.3%)	8,270 (19.6%)	5,429 (24.5%)	**<0.001**
**Past medical history at baseline**				
**Hypertension, n (%)**	47,181 (73.3%)	26,265 (62.2%)	20,916 (94.3%)	**<0.001**
**Hyperlipidemia, n (%)**	43,602 (67.7%)	23,831 (56.4%)	19,771 (89.1%)	**<0.001**
**Chronic Kidney Disease, n (%)**	16,546 (25.7%)	7,254 (17.2%)	9,292 (41.9%)	**<0.001**
**CEV Prior, n (%)**	12,160 (18.9%)	5,989 (14.2%)	6,171 (27.8%)	**<0.001**
**PAD Prior, n (%)**	1,968 (3.1%)	847 (2.0%)	1,121 (5.1%)	**<0.001**
**Atrial Fibrillation Prior, n (%)**	5,404 (8.4%)	2,811 (6.7%)	2,593 (11.7%)	**<0.001**
**MI Prior, n (%)**	2,603 (4.0%)	1,200 (2.8%)	1,403 (6.3%)	**<0.001**
**Ischemic Stroke Prior, n (%)**	2,516 (3.9%)	1,227 (2.9%)	1,289 (5.8%)	**<0.001**
**Heart Failure Prior, n (%)**	5,169 (8.0%)	2,151 (5.1%)	3,018 (13.6%)	**<0.001**
**Outcomes**				
**CEV, n (%)**				**<0.001**
**No**	33,451 (51.9%)	24,817 (58.8%)	8,634 (38.9%)	
**Yes**	30,947 (48.1%)	17,402 (41.2%)	13,545 (61.1%)	
**Had PAD in Claims, n (%)**	8,912 (13.8%)	4,664 (11.0%)	4,248 (19.2%)	
**Had Atrial Fibrillation in Claims, n (%)**	14,123 (22.0%)	7,951 (18.8%)	6,172 (27.8%)	
**Had MI in Claims, n (%)**	8,810 (13.7%)	4,540 (10.8%)	4,270 (19.3%)	
**Had Ischemic Stroke in Claims, n (%)**	8,232 (12.8%)	4,482 (10.6%)	3,750 (16.9%)	
**Had Heart Failure in claims, n (%)**	16,499 (25.6%)	8,311 (19.7%)	8,188 (36.9%)	

[+]Low Socioeconomic Status defined as Yost Index ≤ 2. ADT: Androgen deprivation therapy; AJCC: American Joint Committee on Cancer; *Chi-square test for categorical variables and Mann-Whitney U test for continuous non-normally distributed data; [#]Other marital status includes separated, divorced, and widowed; α**:** Due to the low sensitivity of radiation treatment data from SEER, it cannot be definitively said that a patient did not receive radiotherapy, so "no" and "unknown" categories are combined

Supplementary Table S12 Baseline characteristics and cardiovascular outcomes in non-Hispanic Black subgroup of cohort 1 and enrolled in Medicare parts A and B. The bold font represents statistically significant results.

Non-Hispanic Black	Total	Non-Diabetic	Diabetic	P-value*
**Baseline Characteristics**				
Sample size	16,001	9,523	6,478	
Age at cancer diagnosis (median, IQR)	71 (68-75)	70 (67-74)	72 (69-76)	**<0.001**
Race/Ethnicity, n (%)				
Non-Hispanic White	-	-	-	
Non-Hispanic Black	-	-	-	
Hispanic	-	-	-	
Other	-	-	-	
Marital Status, n (%)				0.31
Single (Never married)	1,805 (11.3%)	1,075 (11.3%)	730 (11.3%)	
Married (Including common law)	6,110 (38.2%)	3,590 (37.7%)	2,520 (38.9%)	
Other	2,192 (13.7%)	1,337 (14.0%)	855 (13.2%)	
Unknown	5,894 (36.8%)	3,521 (37.0%)	2,373 (36.6%)	
Education, n (%)				**<0.001**
High Education	10,204 (63.8%)	5,883 (61.8%)	4,321 (66.7%)	
Low Education	5,797 (36.2%)	3,640 (38.2%)	2,157 (33.3%)	
Socioeconomic Status (SES), n (%)				0.14
High SES	4,022 (25.1%)	2,446 (25.7%)	1,576 (24.3%)	
Low SES	11,452 (71.6%)	6,761 (71.0%)	4,691 (72.4%)	
Patient Rurality, n (%)				0.83
Rural	1,177 (7.4%)	704 (7.4%)	473 (7.3%)	
Urban	14,822 (92.6%)	8,818 (92.6%)	6,004 (92.7%)	
Prostate cancer stage, n (%)				**<0.001**
I	1,633 (10.2%)	933 (9.8%)	700 (10.8%)	
II	4,379 (27.4%)	2,556 (26.8%)	1,823 (28.1%)	
III	372 (2.3%)	247 (2.6%)	125 (1.9%)	
IV	854 (5.3%)	540 (5.7%)	314 (4.8%)	
Unknown	8,763 (54.8%)	5,247 (55.1%)	3,516 (54.3%)	
Grade, n (%)				0.76

**1**	1,625 (10.2%)	963 (10.1%)	662 (10.2%)	
**2**	5,761 (36.0%)	3,460 (36.3%)	2,301 (35.5%)	
**3**	7,039 (44.0%)	4,178 (43.9%)	2,861 (44.2%)	
**4**	32 (0.2%)	17 (0.2%)	15 (0.2%)	
**9**	1,544 (9.6%)	905 (9.5%)	639 (9.9%)	
**Surgery, n (%)**				**0.008**
**No Surgery**	9,176 (78.0%)	5,395 (77.1%)	3,781 (79.2%)	
**Surgery**	2,593 (22.0%)	1,600 (22.9%)	993 (20.8%)	
**Radiotherapy, n (%)**				**<0.001**
**No/Unknown Radiotherapy**[α]	6,806 (56.9%)	4,083 (57.4%)	2,723 (56.2%)	
**Beam Radiation**	3,813 (31.9%)	2,171 (30.5%)	1,642 (33.9%)	
**Implanted Radiation**	531 (4.4%)	343 (4.8%)	188 (3.9%)	
**Other**	809 (6.8%)	513 (7.2%)	296 (6.1%)	
**Chemotherapy, n (%)**	121 (1.0%)	89 (1.3%)	32 (0.7%)	**0.001**
**ADT, n (%)**	3,241 (20.3%)	1,774 (18.6%)	1,467 (22.6%)	**<0.001**
**Past medical history at baseline**				
**Hypertension, n (%)**	12,058 (75.4%)	5,827 (61.2%)	6,231 (96.2%)	**<0.001**
**Hyperlipidemia, n (%)**	9,485 (59.3%)	4,028 (42.3%)	5,457 (84.2%)	**<0.001**
**Chronic Kidney Disease, n (%)**	4,971 (31.1%)	1,907 (20.0%)	3,064 (47.3%)	**<0.001**
**CEV Prior, n (%)**	2,672 (16.7%)	1,023 (10.7%)	1,649 (25.5%)	**<0.001**
**PAD Prior, n (%)**	530 (3.3%)	181 (1.9%)	349 (5.4%)	**<0.001**
**Atrial Fibrillation Prior, n (%)**	745 (4.6%)	271 (2.8%)	474 (7.3%)	**<0.001**
**MI Prior, n (%)**	548 (3.4%)	194 (2.0%)	354 (5.5%)	**<0.001**
**Ischemic Stroke Prior, n (%)**	676 (4.2%)	259 (2.7%)	417 (6.4%)	**<0.001**
**Heart Failure Prior, n (%)**	1,333 (8.3%)	445 (4.7%)	888 (13.7%)	**<0.001**
**Outcomes**				
**CEV, n (%)**				**<0.001**
**No**	8,908 (55.7%)	6,132 (64.4%)	2,776 (42.9%)	
**Yes**	7,093 (44.3%)	3,391 (35.6%)	3,702 (57.1%)	
**Had PAD in Claims, n (%)**	2,130 (13.3%)	908 (9.5%)	1,222 (18.9%)	
**Had Atrial Fibrillation in Claims, n (%)**	2,362 (%)	1,111 (%)	1,251 (%)	
**Had MI in Claims, n (%)**	2,074 (13.0%)	899 (9.4%)	1,175 (18.1%)	

**Had Ischemic Stroke in Claims, n (%)**	2,161 (13.5%)	958 (10.1%)	1,203 (18.6%)
**Had Heart Failure in claims, n (%)**	4,026 (25.2%)	1,751 (18.4%)	2,275 (35.1%)

ADT: Androgen deprivation therapy; AJCC: American Joint Committee on Cancer; *Chi-square test for categorical variables and Mann-Whitney U test for continuous non-normally distributed data; #Other marital status includes separated, divorced, and widowed; α**:** Due to the low sensitivity of radiation treatment data from SEER, it cannot be definitively said that a patient did not receive radiotherapy, so "no" and "unknown" categories are combined

Supplementary Table S13 Baseline characteristics and cardiovascular outcomes in low education subgroup of cohort 2 and enrolled in Medicare parts A, B and D. The bold font represents statistically significant results.

Low Education+	Total	No DM	DM-Metformin	DM-Others	P-value*
**Baseline Characteristics**					
**Sample size**	6,840	5,565	1,087	188	
**Age at cancer diagnosis (median, IQR)**	73 (69-78)	73 (69-78)	72 (69-77)	74 (70-79)	0.019
**Race/Ethnicity, n (%)**					**<0.001**
**Non-Hispanic White**	5,281 (77.2%)	4,453 (80.0%)	715 (65.8%)	113 (60.1%)	
**Non-Hispanic Black**	559 (8.2%)	419 (7.5%)	111 (10.2%)	29 (15.4%)	
**Hispanic**	540 (7.9%)	367 (6.6%)	145 (13.3%)	28 (14.9%)	
**Other**	460 (6.7%)	326 (5.9%)	116 (10.7%)	18 (9.6%)	
**Marital Status, n (%)**					0.65
**Single (Never married)**	540 (7.9%)	448 (8.1%)	76 (7.0%)	16 (8.5%)	
**Married (Including common law)**	3,236 (47.3%)	2,625 (47.2%)	532 (48.9%)	79 (42.0%)	
**Other**	627 (9.2%)	511 (9.2%)	97 (8.9%)	19 (10.1%)	
**Unknown**	2,437 (35.6%)	1,981 (35.6%)	382 (35.1%)	74 (39.4%)	
**Education, n (%)**					
**High Education**	-	-	-	-	
**Low Education**	-	-	-	-	
**Socioeconomic Status (SES), n (%)**					**0.001**
**High SES**	4,025 (58.8%)	3,333 (59.9%)	602 (55.4%)	90 (47.9%)	

**Low SES**	2,418 (35.4%)	1,917 (34.4%)	419 (38.5%)	82 (43.6%)	
**Unknown**	397 (5.8%)	315 (5.7%)	66 (6.1%)	16 (8.5%)	
**Patient Rurality, n (%)**					0.36
**Rural**	208 (3.0%)	>175 (>3.0%)	27 (2.5%)	<11	
**Urban**	6,631 (97.0%)	>5,385 (>96.5%)	1,060 (97.5%)	>180 (>97.5%)	
**Prostate cancer stage, n (%)**					0.42
**I**	138 (2.0%)	>115 (>2.0%)	15 (1.4%)	<11	
**II**	1,231 (18.0%)	989 (17.8%)	207 (19.0%)	35 (18.6%)	
**III**	379 (5.5%)	>315 (>5.5%)	51 (4.7%)	<11	
**IV**	1,047 (15.3%)	841 (15.1%)	170 (15.6%)	36 (19.1%)	
**Unknown**	4,045 (59.1%)	3,296 (59.2%)	644 (59.2%)	105 (55.9%)	
**Grade, n (%)**					0.48
**1**	186 (2.7%)	148 (2.7%)	>30 (>2.5%)	<11	
**2**	1,251 (18.3%)	1,037 (18.6%)	181 (16.7%)	33 (17.6%)	
**3**	4,464 (65.3%)	3,601 (64.7%)	742 (68.3%)	121 (64.4%)	
**4**	18 (0.3%)	16 (0.3%)	<11	<11	
**9**	921 (13.5%)	763 (13.7%)	131 (12.1%)	27 (14.4%)	
**Surgery, n (%)**					0.10
**No Surgery**	3,620 (76.3%)	2,923 (75.8%)	591 (77.9%)	106 (82.8%)	
**Surgery**	1,124 (23.7%)	934 (24.2%)	168 (22.1%)	22 (17.2%)	
**Radiotherapy, n (%)**					0.72
**No/Unknown Radiotherapy**[α]	2,541 (53.1%)	2,081 (53.6%)	388 (50.5%)	72 (55.4%)	
**Beam Radiation**	1,744 (36.5%)	1,401 (36.1%)	298 (38.8%)	45 (34.6%)	
**Implanted Radiation**	90 (1.9%)	>75 (>1.8%)	12 (1.6%)	<11	
**Other**	409 (8.5%)	>325 (>8.0%)	70 (9.1%)	11 (8.5%)	
**Chemotherapy, n (%)**	298 (6.2%)	>235 (>6.0%)	51 (6.6%)	<11	0.82
**ADT, n (%)**	6,840 (100.0%)	5,565 (100.0%)	1,087 (100.0%)	188 (100.0%)	-
**Past medical history at baseline**					
**Hypertension, n (%)**	5,058 (73.9%)	3,961 (71.2%)	932 (85.7%)	165 (87.8%)	<0.001

**Hyperlipidemia, n (%)**	4,913 (71.8%)	3,848 (69.1%)	911 (83.8%)	154 (81.9%)	<0.001
**Chronic Kidney Disease, n (%)**	1,850 (27.0%)	1,350 (24.3%)	388 (35.7%)	112 (59.6%)	<0.001
**CEV Prior, n (%)**	1,502 (22.0%)	1,179 (21.2%)	257 (23.6%)	66 (35.1%)	<0.001
**PAD Prior, n (%)**	220 (3.2%)	168 (3.0%)	41 (3.8%)	11 (5.9%)	0.051
**Atrial Fibrillation Prior, n (%)**	785 (11.5%)	628 (11.3%)	125 (11.5%)	32 (17.0%)	0.053
**MI Prior, n (%)**	311 (4.5%)	230 (4.1%)	65 (6.0%)	16 (8.5%)	<0.001
**Ischemic Stroke Prior, n (%)**	308 (4.5%)	233 (4.2%)	61 (5.6%)	14 (7.4%)	0.017
**Heart Failure Prior, n (%)**	553 (8.1%)	423 (7.6%)	98 (9.0%)	32 (17.0%)	<0.001
**Outcomes**					
**CEV, n (%)**					<0.001
**No**	3,326 (48.6%)	2,793 (50.2%)	474 (43.6%)	59 (31.4%)	
**Yes**	3,514 (51.4%)	2,772 (49.8%)	613 (56.4%)	129 (68.6%)	
**Had PAD in Claims, n (%)**	993 (14.5%)	761 (13.7%)	190 (17.5%)	42 (22.3%)	<0.001
**Had Atrial Fibrillation in Claims, n (%)**	1,747 (25.6%)	1,412 (25.4%)	273 (25.1%)	62 (33.0%)	0.059
**Had MI in Claims, n (%)**	995 (14.5%)	738 (13.3%)	212 (19.5%)	45 (23.9%)	<0.001
**Had Ischemic Stroke in Claims, n (%)**	911 (13.3%)	704 (12.7%)	174 (16.0%)	33 (17.6%)	0.003
**Had Heart Failure in claims, n (%)**	1,751 (25.6%)	1,332 (23.9%)	338 (31.1%)	81 (43.1%)	<0.001

[+]Low Education defined as high school education <25%. ADT: Androgen deprivation therapy; AJCC: American Joint Committee on Cancer; DM: diabetes; *Chi-square test for categorical variables and Mann-Whitney U test for continuous non-normally distributed data; #Other marital status includes separated, divorced, and widowed; ^Other medications include alpha-glucosidase inhibitors, amylin analogs, dipeptidyl peptidase-4 (DPP-4), insulin, meglitinides, sulfonylureas, thiazolidinedione; α**:** Due to the low sensitivity of radiation treatment data from SEER, it cannot be definitively said that a patient did not receive radiotherapy, so "no" and "unknown" categories are combined

Supplementary Table S14 Baseline characteristics and cardiovascular outcomes in low socio-economic status subgroup of cohort 2 and enrolled in Medicare parts A, B and D. The bold font represents statistically significant results.

Low Socio-Economic Status+	Total	No DM	DM-Metformin	DM-Others	P-value*
**Baseline Characteristics**					
**Sample size**	6,405	5,067	1,077	261	
**Age at cancer diagnosis (median, IQR)**	73 (69-78)	73 (69-78)	72 (69-77)	73 (69-79)	**<0.001**
**Race/Ethnicity, n (%)**					**<0.001**
**Non-Hispanic White**	4,455 (69.6%)	3,680 (72.6%)	642 (59.6%)	133 (51.0%)	
**Non-Hispanic Black**	1,042 (16.3%)	778 (15.4%)	192 (17.8%)	72 (27.6%)	
**Hispanic**	606 (9.5%)	396 (7.8%)	170 (15.8%)	40 (15.3%)	
**Other**	302 (4.7%)	213 (4.2%)	73 (6.8%)	16 (6.1%)	
**Marital Status, n (%)**					0.93
**Single (Never married)**	632 (9.9%)	500 (9.9%)	104 (9.7%)	28 (10.7%)	
**Married (Including common law)**	2,552 (39.8%)	2,028 (40.0%)	426 (39.6%)	98 (37.5%)	
**Other**	772 (12.1%)	617 (12.2%)	122 (11.3%)	33 (12.6%)	
**Unknown**	2,449 (38.2%)	1,922 (37.9%)	425 (39.5%)	102 (39.1%)	
**Education, n (%)**					0.079
**High Education**	3,987 (62.2%)	3,150 (62.2%)	658 (61.1%)	179 (68.6%)	
**Low Education**	2,418 (37.8%)	1,917 (37.8%)	419 (38.9%)	82 (31.4%)	
**Socioeconomic Status (SES), n (%)**					
**High SES**	-	-	-	-	
**Low SES**	-	-	-	-	
**Unknown**	-	-	-	-	
**Patient Rurality, n (%)**					**0.025**
**Rural**	1,612 (25.2%)	1,309 (25.8%)	252 (23.4%)	51 (19.5%)	
**Urban**	4,793 (74.8%)	3,758 (74.2%)	825 (76.6%)	210 (80.5%)	
**Prostate cancer stage, n (%)**					0.68
**I**	164 (2.6%)	>130 (>2.5%)	26 (2.4%)	<11	
**II**	1,204 (18.8%)	941 (18.6%)	206 (19.1%)	57 (21.8%)	
**III**	291 (4.5%)	225 (4.4%)	54 (5.0%)	12 (4.6%)	

**IV**	988 (15.4%)	802 (15.8%)	155 (14.4%)	31 (11.9%)	
**Unknown**	3,758 (58.7%)	>2,965 (>58.5%)	636 (59.1%)	>150 (58.5%)	
**Grade, n (%)**					0.14
**1**	184 (2.9%)	141 (2.8%)	32 (3.0%)	11 (4.2%)	
**2**	1,115 (17.4%)	859 (17.0%)	210 (19.5%)	46 (17.6%)	
**3**	4,098 (64.0%)	3,251 (64.2%)	>685 (>64.0%)	>155 (>60.0%)	
**4**	28 (0.4%)	24 (0.5%)	<11	<11	
**9**	980 (15.3%)	792 (15.6%)	141 (13.1%)	47 (18.0%)	
**Surgery, n (%)**					0.069
**No Surgery**	3,516 (80.2%)	2,771 (79.6%)	593 (81.2%)	152 (86.4%)	
**Surgery**	869 (19.8%)	708 (20.4%)	137 (18.8%)	24 (13.6%)	
**Radiotherapy, n (%)**					**0.049**
**No/Unknown Radiotherapy**[α]	2,474 (55.6%)	1,982 (56.2%)	389 (52.4%)	103 (57.5%)	
**Beam Radiation**	1,589 (35.7%)	1,230 (34.9%)	289 (38.9%)	70 (39.1%)	
**Implanted Radiation**	80 (1.8%)	>60 (>2.0%)	11 (1.5%)	<11	
**Other**	304 (6.8%)	>240 (>6.5%)	54 (7.3%)	<11	
**Chemotherapy, n (%)**	251 (5.6%)	>200 (>5.5%)	41 (5.5%)	<11	0.77
**ADT, n (%)**	6,405 (100.0%)	5,067 (100.0%)	1,077 (100.0%)	261 (100.0%)	-
**Past medical history at baseline**					
**Hypertension, n (%)**	4,989 (77.9%)	3,792 (74.8%)	955 (88.7%)	242 (92.7%)	**<0.001**
**Hyperlipidemia, n (%)**	4,445 (69.4%)	3,339 (65.9%)	887 (82.4%)	219 (83.9%)	**<0.001**
**Chronic Kidney Disease, n (%)**	1,984 (31.0%)	1,431 (28.2%)	397 (36.9%)	156 (59.8%)	**<0.001**
**CEV Prior, n (%)**	1,481 (23.1%)	1,099 (21.7%)	282 (26.2%)	100 (38.3%)	**<0.001**
**PAD Prior, n (%)**	267 (4.2%)	185 (3.7%)	58 (5.4%)	24 (9.2%)	**<0.001**
**Atrial Fibrillation Prior, n (%)**	666 (10.4%)	501 (9.9%)	124 (11.5%)	41 (%)	**0.005**
**MI Prior, n (%)**	323 (5.0%)	232 (4.6%)	62 (5.8%)	29 (11.1%)	**<0.001**
**Ischemic Stroke Prior, n (%)**	329 (5.1%)	237 (4.7%)	68 (6.3%)	24 (9.2%)	**<0.001**
**Heart Failure Prior, n (%)**	626 (9.8%)	440 (8.7%)	126 (11.7%)	60 (23.0%)	**<0.001**
**Outcomes**					
**CEV, n (%)**					**<0.001**

**No**	2,838 (44.3%)	2,358 (46.5%)	413 (38.3%)	67 (25.7%)	
**Yes**	3,567 (55.7%)	2,709 (53.5%)	664 (61.7%)	194 (74.3%)	
**Had PAD in Claims, n (%)**	1,078 (16.8%)	787 (15.5%)	219 (20.3%)	72 (27.6%)	**<0.001**
**Had Atrial Fibrillation in Claims, n (%)**	1,599 (%)	1,209 (%)	296 (%)	94 (%)	**<0.001**
**Had MI in Claims, n (%)**	1,028 (16.0%)	754 (14.9%)	201 (18.7%)	73 (28.0%)	**<0.001**
**Had Ischemic Stroke in Claims, n (%)**	946 (14.8%)	705 (13.9%)	184 (17.1%)	57 (21.8%)	**<0.001**
**Had Heart Failure in claims, n (%)**	1,918 (29.9%)	1,383 (27.3%)	397 (36.9%)	138 (52.9%)	**<0.001**

$^{+}$Low Socioeconomic Status defined as Yost Index ≤ 2. ADT: Androgen deprivation therapy; AJCC: American Joint Committee on Cancer; DM: diabetes; *Chi-square test for categorical variables and Mann-Whitney U test for continuous non-normally distributed data; #Other marital status includes separated, divorced, and widowed; ^Other medications include alpha-glucosidase inhibitors, amylin analogs, dipeptidyl peptidase-4 (DPP-4), insulin, meglitinides, sulfonylureas, thiazolidinedione; α**:** Due to the low sensitivity of radiation treatment data from SEER, it cannot be definitively said that a patient did not receive radiotherapy, so "no" and "unknown" categories are combined

Supplementary Table S15 Baseline characteristics and cardiovascular outcomes in non-Hispanic Black subgroup of cohort 2 and enrolled in Medicare parts A, B and D. The bold font represents statistically significant results.

Non-Hispanic Black	Total	No DM	DM-Metformin	DM-Others	P-value*
**Baseline Characteristics**					
**Sample size**	1,436	1,069	276	91	
**Age at cancer diagnosis (median, IQR)**	71 (68-76)	71 (68-76)	70 (68-74)	71 (68-76)	**0.032**
**Race/Ethnicity, n (%)**					
**Non-Hispanic White**	-	-	-	-	
**Non-Hispanic Black**	-	-	-	-	
**Hispanic**	-	-	-	-	
**Other**	-	-	-	-	
**Marital Status, n (%)**					0.11
**Single (Never married)**	231 (16.1%)	>170 (>16.0%)	45 (16.3%)	>12 (>14.0%)	

**Married (Including common law)**	466 (32.5%)	352 (32.9%)	83 (30.1%)	31 (34.1%)	
**Other**	229 (15.9%)	>180 (>17.0%)	35 (12.7%)	<11	
**Unknown**	510 (35.5%)	359 (33.6%)	113 (40.9%)	38 (41.8%)	
**Education, n (%)**					0.34
**High Education**	877 (61.1%)	650 (60.8%)	165 (59.8%)	62 (68.1%)	
**Low Education**	559 (38.9%)	419 (39.2%)	111 (40.2%)	29 (31.9%)	
**Socioeconomic Status (SES), n (%)**					0.42
**High SES**	314 (21.9%)	>230 (>21.5%)	69 (25.0%)	>12 (>15.0%)	
**Low SES**	1,042 (72.6%)	778 (72.8%)	192 (69.6%)	72 (79.1%)	
**Unknown**	80 (5.6%)	>55 (>5.5%)	15 (5.4%)	<11	
**Patient Rurality, n (%)**					**0.048**
**Rural**	119 (8.3%)	79 (7.4%)	33 (12.0%)	7 (7.7%)	
**Urban**	1,317 (91.7%)	990 (92.6%)	243 (88.0%)	84 (92.3%)	
**Prostate cancer stage, n (%)**					**0.009**
**I**	34 (2.4%)	25 (2.3%)	<11	<11	
**II**	320 (22.3%)	228 (21.3%)	73 (26.4%)	19 (20.9%)	
**III**	50 (3.5%)	35 (3.3%)	<11	<11	
**IV**	241 (16.8%)	205 (19.2%)	24 (8.7%)	12 (13.2%)	
**Unknown**	791 (55.1%)	576 (53.9%)	163 (59.1%)	52 (57.1%)	
**Grade, n (%)**					0.19
**1**	>40 (>2.5%)	>25 (>2.5%)	<11	<11	
**2**	267 (18.6%)	184 (17.2%)	66 (23.9%)	17 (18.7%)	
**3**	921 (64.1%)	693 (64.8%)	172 (62.3%)	56 (61.5%)	
**4**	<11	<11	<11	<11	
**9**	200 (13.9%)	158 (14.8%)	27 (9.8%)	15 (16.5%)	
**Surgery, n (%)**					0.54
**No Surgery**	868 (85.0%)	656 (84.5%)	159 (85.5%)	53 (89.8%)	
**Surgery**	153 (15.0%)	>115 (>15.0%)	27 (14.5%)	<11	

**Radiotherapy, n (%)**					0.22
**No/Unknown Radiotherapy[α]**	598 (57.7%)	468 (59.5%)	96 (50.5%)	34 (56.7%)	
**Beam Radiation**	351 (33.9%)	253 (32.2%)	74 (38.9%)	24 (40.0%)	
**Implanted Radiation**	16 (1.5%)	>11 (>1.5%)	<11	<11	
**Other**	71 (6.9%)	52 (6.6%)	17 (8.9%)	<11	
**Chemotherapy, n (%)**	55 (5.3%)	50 (6.4%)	<11	<11	**0.014**
**ADT, n (%)**	1,436 (100.0%)	1,069 (100.0%)	276 (100.0%)	91 (100.0%)	-
**Past medical history at baseline**					
**Hypertension, n (%)**	1,191 (82.9%)	856 (80.1%)	252 (91.3%)	83 (91.2%)	**<0.001**
**Hyperlipidemia, n (%)**	841 (58.6%)	575 (53.8%)	200 (72.5%)	66 (72.5%)	**<0.001**
**Chronic Kidney Disease, n (%)**	559 (38.9%)	398 (37.2%)	101 (36.6%)	60 (65.9%)	**<0.001**
**CEV Prior, n (%)**	292 (20.3%)	197 (18.4%)	59 (21.4%)	36 (39.6%)	**<0.001**
**PAD Prior, n (%)**	64 (4.5%)	>40 (>4.0%)	12 (4.3%)	<11	**0.12**
**Atrial Fibrillation Prior, n (%)**	72 (5.0%)	45 (4.2%)	16 (%)	11 (%)	**0.003**
**MI Prior, n (%)**	67 (4.7%)	>40 (>3.5%)	15 (5.4%)	<11	**0.007**
**Ischemic Stroke Prior, n (%)**	85 (5.9%)	>60 (>5.5%)	18 (6.5%)	<11	**0.65**
**Heart Failure Prior, n (%)**	142 (9.9%)	90 (8.4%)	27 (9.8%)	25 (27.5%)	**<0.001**
**Outcomes**					
**CEV, n (%)**					**<0.001**
**No**	676 (47.1%)	540 (50.5%)	112 (40.6%)	24 (26.4%)	
**Yes**	760 (52.9%)	529 (49.5%)	164 (59.4%)	67 (73.6%)	
**Had PAD in Claims, n (%)**	248 (17.3%)	171 (16.0%)	56 (20.3%)	21 (23.1%)	0.077
**Had Atrial Fibrillation in Claims, n (%)**	232 (%)	168 (%)	39 (%)	25 (%)	**0.008**
**Had MI in Claims, n (%)**	222 (15.5%)	153 (14.3%)	41 (14.9%)	28 (30.8%)	**<0.001**
**Had Ischemic Stroke in Claims, n (%)**	230 (16.0%)	159 (14.9%)	54 (19.6%)	17 (18.7%)	0.13
**Had Heart Failure in claims, n (%)**	413 (28.8%)	270 (25.3%)	91 (33.0%)	52 (57.1%)	**<0.001**

ADT: Androgen deprivation therapy; AJCC: American Joint Committee on Cancer; DM: diabetes; *Chi-square test for categorical variables and Mann-Whitney U test for continuous non-normally distributed data; [#]Other marital status includes separated, divorced,

and widowed; ^Other medications include alpha-glucosidase inhibitors, amylin analogs, dipeptidyl peptidase-4 (DPP-4), insulin, meglitinides, sulfonylureas, thiazolidinedione; α**:** Due to the low sensitivity of radiation treatment data from SEER, it cannot be definitively said that a patient did not receive radiotherapy, so "no" and "unknown" categories are combined

Supplementary Table S16 Baseline characteristics of the excluded patients

Non-Hispanic Black	Total	No DM	DM	P-value*
**Baseline Characteristics**				
**Sample size**	5108	3563	1545	
**Age at cancer diagnosis (median, IQR)**	72 (68-77)	71 (68-76)	74 (70-78)	<0.001
**Race/Ethnicity, n (%)**				
**Non-Hispanic White**	505 (9.9%)	364 (10.2%)	141 (9.1%)	
**Non-Hispanic Black**	65 (1.3%)	40 (1.1%)	25 (1.6%)	
**Hispanic**	69 (1.4%)	46 (1.3%)	23 (1.5%)	
**Other**	42 (0.8%)	25 (0.7%)	17 (1.1%)	
**Unknown Race (%)**	4427 (86.7%)	3088 (86.7%)	1339 (86.7%)	
**Maried, n (%)**	928 (18.2%)	646 (18.1%)	282 (18.3%)	0.99
**Low SES**	1857 (36.4%)	1245 (34.9%)	612 (39.6%)	0.006
**Low Education**	2894 (56.7%)	2052 (57.6%)	842 (54.5%)	0.040
**Surgery (%)**	256 (7.0%)	182 (7.3%)	74 (6.4%)	0.32
**Radiotherapy (%)**	-	-	-	0.12
**ADT use (%)**	690 (13.5%)	425 (11.9%)	265 (17.2%)	<0.001
**Chemotherapy use (%)**	-	-	-	0.91

**PC Stage III/IV (%)**	Very low (~1.6%)	-	-	0.95
**Grade 3 (%)**	1436 (28.1%)	962 (27.0%)	474 (30.7%)	0.007
**Hypertension (%)**	3530 (69.1%)	2093 (58.7%)	1437 (93.0%)	<0.001
**Hyperlipidemia (%)**	3523 (69.0%)	2107 (59.1%)	1416 (91.7%)	<0.001
**Chronic Kidney Disease (%)**	1013 (19.8%)	448 (12.6%)	565 (36.6%)	<0.001
**Prior CV Event (%)**	808 (15.8%)	437 (12.3%)	371 (24.0%)	<0.001

$^{+}$Low Socioeconomic Status defined as Yost Index ≤ 2. ADT: Androgen deprivation therapy; AJCC: American Joint Committee on Cancer; *Chi-square test for categorical variables and Mann-Whitney U test for continuous non-normally distributed data; $^{\#}$Other marital status includes separated, divorced, and widowed; α**:** Due to the low sensitivity of radiation treatment data from SEER, it cannot be definitively said that a patient did not receive radiotherapy, so "no" and "unknown" categories are combined. – Some numbers are hidden according to the Centers for Medicare & Medicaid Services Cell Suppression Policy for data usage.

Supplemental Figures

Supplemental Figure S1 CONSORT Flow Diagram

CONSORT Flow Diagram

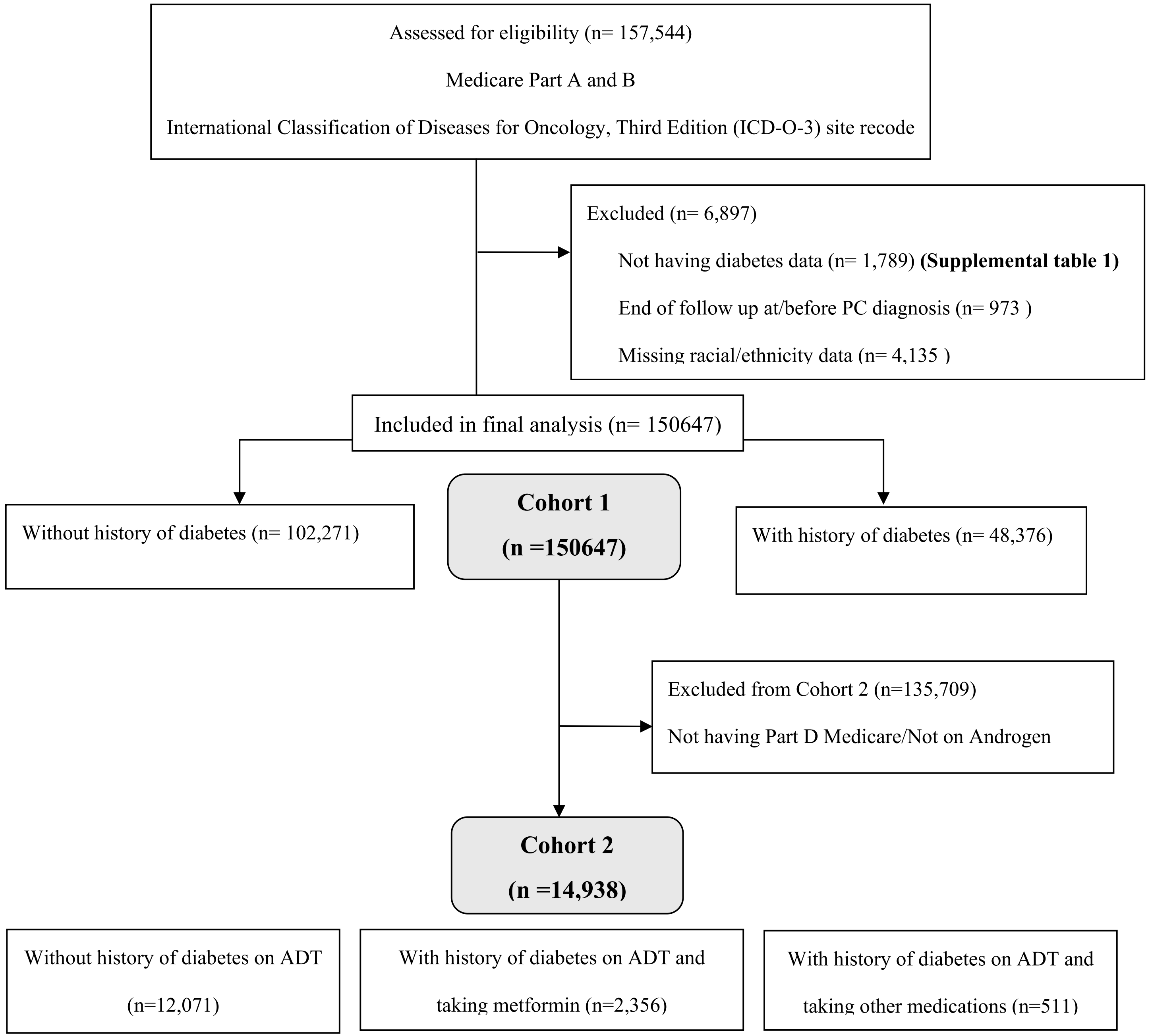

Supplement: Supplementary file 1 [file cancers-17-02854-s001.zip › cancers-3810630-supplementary.pdf]
